# Causal effect of thyroid function on cortical brain structure: A two-sample Mendelian randomization study

**DOI:** 10.1016/j.ibneur.2026.06.010

**Published:** 2026-06-15

**Authors:** Jianxin Shi, Jianhuai Chen, Ruifeng Wang, Heng Zhao

**Affiliations:** a Department of Dermatology, Jiangsu Province Hospital of Chinese Medicine, Affiliated Hospital of Nanjing University of Chinese Medicine, Nanjing, China; b Department of Andrology, Jiangsu Province Hospital of Chinese Medicine, Affiliated Hospital of Nanjing University of Chinese Medicine, Nanjing, China; c Department of Endocrinology, Jinling Hospital, Affiliated Hospital of Medical School, Nanjing University, Nanjing, China

**Keywords:** Thyroid function, Mendelian randomization, Brain structure, Cerebral cortex, Autoimmune diseases

## Abstract

**Background:**

The causal relationship between variations in thyroid dysfunction and brain structure remains elusive. We intended to probe into this causal connection using a Mendelian randomization (MR) approach.

**Methods:**

Aggregated statistics from the genome-wide Association Study (GWAS) on Autoimmune thyroid dysfunction were obtained from the FinnGen database, including autoimmune hyperthyroidism (N = 2,190) and autoimmune hypothyroidism (N = 49,055). Aggregate statistics on cortical area and thickness for GWAS were obtained from the Enhancing NeuroImaging Genetics through Meta-Analysis (ENIGMA) Consortium (N = 51,665). The main method employed is inverse variance weighting (IVW), supplemented by weighted median, Mr-Egger regression, MR-pleotropy residuals, and outliers. Some sensitivity analyses were conducted to determine heterogeneity and pleiotropy.

**Results:**

For autoimmune hypothyroidism (exposure), significant causal associations were observed with cortical thickness (outcome type) in the following regions: pars triangularis (β = −0.003, 95% CI: −0.005 to −0.001, PTH = 0.023; PTH: P-value for cortical thickness,), posterior cingulate (β = 0.003, 95% CI: 0.000–0.006, PTH = 0.034), and transverse temporal (β = 0.006, 95% CI: 0.002–0.010, PTH = 0.004; β = 0.006, 95% CI: 0.001–0.011, PTH = 0.010). For autoimmune hyperthyroidism (exposure), significant causal associations were observed with cortical surface area (outcome type) in the entorhinal (β = −2.576, 95% CI: −4.714 to −0.437, PSA = 0.018; PSA: P-value for cortical surface area), frontal pole (β = −1.195, 95% CI: −2.118 to −0.271, PSA = 0.011), lateral occipital (β = −15.787, 95% CI: −31.171 to −0.403, PSA = 0.044), lingual (β = −10.778, 95% CI: −20.888 to −0.668, PSA = 0.037), postcentral (β = −16.360, 95% CI: −28.410 to −4.309, PSA = 0.008), precentral (β = −16.089, 95% CI: −29.281 to −2.897, PSA = 0.017), and superior parietal (with global weighting; β = 11.360, 95% CI: 0.435–22.285, PSA = 0.042). Additionally, autoimmune hyperthyroidism was causally associated with cortical thickness (outcome type) in the entorhinal (β = 0.009, 95% CI: 0.000–0.018, PTH = 0.039) and posterior cingulate (β = 0.004, 95% CI: 0.001–0.008, PTH = 0.025). These findings remained reliable in the sensitivity analyses.

**Conclusions:**

These findings suggest that autoimmune hyperthyroidism and autoimmune hypothyroidism may have potential causal effects on the surface area and thickness of specific regions of the cerebral cortex, but further studies are needed to fully understand the nature of this relationship.

## 1 Introduction

Thyroid dysfunction is a common clinical condition and thyroid hormone receptors are widely distributed in brain regions including the hippocampus, prefrontal cortex, and thalamus, where they play important roles in regulating brain function. Decreased maternal T4 has been shown to disrupt normal cortical layer-specific migration of fetal neurons in experimental models(de Escobar, et al.,2004). Therefore, abnormal thyroid function may disrupt the normal development and maintenance of brain structure and function, leading to cognitive, emotional, and behavioral disturbances. However, previous observational studies on this topic have been limited by small sample sizes, confounding factors (e.g., sociodemographic characteristics, depressive syndrome, cerebrovascular events), and insufficient statistical robustness.

Despite compelling evidence from animal models and observational studies suggesting that thyroid hormones influence brain architecture, translating these findings into definitive causal understanding in humans has been challenging due to fundamental methodological constraints. Key limitations include temporal dissociation (neuropsychiatric symptoms often precede biochemical diagnosis), the cumulative nature of lifelong thyroid hormone exposure that single time-point measurements cannot capture, and the fluctuating disease activity characteristic of autoimmune thyroid disorders. Therefore, moving beyond association to causation requires an analytical framework—such as Mendelian randomization—that can account for lifelong genetic predisposition to thyroid dysfunction and circumvent biases inherent in conventional observational designs.

Mendelian randomization (MR), which uses genetic variation as an exposure to assess causal relationships with disease-related risk factors, can overcome inherent confounding in observational studies(Davey Smith and Hemani,2014; Ye, et al.,2023). Currently, some associations between thyroid hormones and certain brain regions have been reported, but the causal relationships between thyroid function and cortical structure of the brain have not been confirmed. As an extension of the MR method, two-sample MR analysis allows the use of summary statistics from genome-wide association studies (GWAS) for MR studies without directly analyzing individual-level data. Based on large publicly available GWAS data, we used two-sample MR analysis to illustrate the impact of thyroid function on cortical structure of the brain.

In recent years, with the advancement of neuroimaging technology, an increasing number of studies have focused on the relationship between thyroid dysfunction and changes in brain structure. Cross-sectional studies have shown that patients with hypothyroidism often exhibit structural alterations such as reduced hippocampal volume and thinning of the prefrontal cortex, whereas patients with hyperthyroidism may display different patterns, including decreased total brain volume and increased local cortical thickness. These structural changes are believed to be closely related to the direct regulatory effects of thyroid hormones on neurons, involving various pathways such as modulating neurotrophic factor expression, influencing synaptic plasticity, and regulating neurotransmitter systems. However, a review of the existing literature reveals that most of these studies are small-scale observational studies with sample sizes typically ranging from dozens to hundreds of participants, and significant heterogeneity in study design. More importantly, these observational studies cannot determine whether thyroid dysfunction is the cause of brain structural changes or whether both are influenced by other confounding factors. For example, thyroid diseases are often accompanied by psychiatric symptoms such as depression and anxiety, which themselves are associated with alterations in brain structure. Additionally, multiple covariates—including age, gender, education level, and cardiovascular risk factors—may simultaneously affect thyroid function and brain structure, thereby limiting the causal inference capabilities of traditional observational studies.

MR, as an emerging causal inference tool, offers a novel approach to addressing the aforementioned research challenges. This method, based on Mendelian inheritance laws, uses genotypes as instrumental variables to infer causal relationships between exposure factors and outcomes. Since genotypes are randomly assigned during gamete formation and are not influenced by common confounding factors such as postnatal environmental factors, lifestyle, or disease status, MR can effectively avoid confounding biases and reverse causation issues prevalent in traditional epidemiological studies. Particularly in the context of the thyroid-brain axis investigated in this study, the MR method can mitigate the effects of comorbidities commonly observed in thyroid disease patients (e.g., cardiovascular diseases, metabolic syndrome) on brain structure, providing more reliable causal evidence. In recent years, with the rapid development of large-scale biobanks and GWAS studies—especially the summary statistics provided by large consortia such as FinnGen and Enhancing NeuroImaging Genetics through Meta-Analysis (ENIGMA)—rich data resources have become available for conducting two-sample MR studies. These resources not only offer large sample sizes and high statistical power but also ensure data reliability through stringent quality control and standardized procedures. Against this backdrop, this study systematically applies the MR method for the first time to explore the causal impact of autoimmune thyroid dysfunction on brain cortical structure, filling a gap in causal evidence in this field.

We utilized human genetic data within the MR framework to reveal the influences of thyroid function on cortical structure of the brain, defined as surface area and cortical thickness of the human brain cortex detected by MRI. Two sets of parameters: autoimmune hyperthyroidism and autoimmune hypothyroidism were used to provide MR estimates. We also conducted subgroup analyses based on different brain regions. These results might provide new insights into the potential impacts of thyroid function on cortical structure of the brain.

Specifically, this study aimed to address two primary research questions: (1) Does genetically predicted thyroid function causally affect cortical thickness, cortical surface area, or both? (2) Do the patterns of causal effects differ between autoimmune hyperthyroidism and autoimmune hypothyroidism? By answering these questions, our findings may provide new insights into the potential causal impacts of thyroid dysfunction on cortical brain structure.

## 2 Methods

### 2.1 Thyroid function

We utilized summaries of data from the FinnGen Consortium (https://r11.finngen.fi/).The Thyroid function GWAS data were obtained from FinnGen database. FinnGen database is a genomics project in Finland aimed at creating a comprehensive reference dataset of the Finnish population's genome. The project has collected genotypic and clinical data from over 500,000 Finns and is one of the largest single ethnic group genomics projects in the world. GWAS compliant dataset was found in the FinnGen database. The GWAS of Hypothyroidism(strict autoimmune) included 1 cohorts of European ancestry (n = 329,775; 49,055 cases and 280,720 controls), the GWAS of Hyperthyroidism(autoimmune) included 1 cohorts of European ancestry (n = 336,787; 2190cases and 364,597 controls)(Kurki, et al.,2023).

### 2.2 Brain cortical structure

The brain cortical structure-related GWAS data were obtained from the ENIGMA Consortium(Grasby, et al.,2020). The brain surface area and cortical thickness were measured in 51,665 individuals, primarily (∼94%) of European descent across 60 cohorts around the world, using MRI. Meta results including only European-ancestry participants were used in our study (detailed cohort information was listed in Supplementary Table S3). The 34 regions were defined based on the Desikan-Killiany atlas, establishing coarse partitions of the cortex, and the regional boundaries were determined according to the gyral anatomy labelled between the depths of the sulci(Desikan, et al.,2006). The regions were averaged between both hemispheres. We performed MR analysis from hypothyroidism on surface area and cortical thickness of the entire cortex, as well as surface area and cortical thickness for 34 brain cortical regions with known functional specialisations with or without the weighted estimates of the entire brain, yielding 138 outcomes. Data comprising global weighted estimates indicated the surface area and cortical thickness of specific regions across the surface area and cortical thickness of the entire brain, while those without global weighted estimates indicated the surface area and cortical thickness measure of specific regions, regardless of the total brain surface area and cortical thickness.

### 2.3 Selection of instrumental variables

We selected appropriate instrumental variables from the GWAS data following the steps below. First, we screened for SNPs with a significance threshold of P-value < 5 × 10⁻⁸. Subsequently, we applied linkage disequilibrium (LD) pruning based on the reference panel detailed in Supplementary Table S3, with clumping parameters set to a physical distance of 1000 kb and an r² < 0.001. This stringent threshold, based on established methodological literature in the MR field, ensures that the included SNPs are approximately independent, minimizing potential bias from LD.

Subsequently, we excluded instrumental variables with an F-statistic < 10. The F-statistic was calculated using the formula [F = R^2^× (N-K−1)/K× (1-R^2^)] and this threshold was applied to exclude weak instrument bias (Hu et al., 2022).

**Fig. 1:**
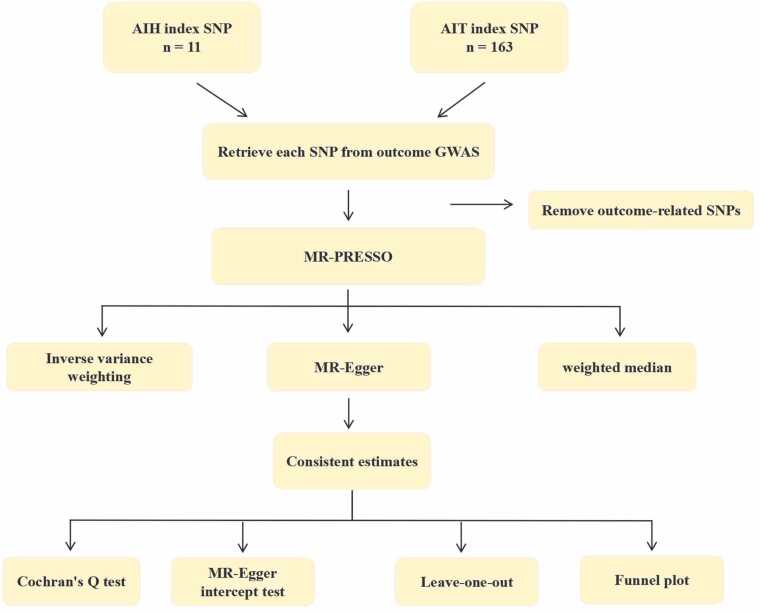
Study flame chart of the Mendelian randomization study revealing the causal relationship between Autoimmune Hyperthyroidism and Autoimmune Hypothyroidism, and the brain cortical structure as defined by the surficial area and thickness of the brain cortex measured using magnetic resonance imaging. SA: Surficial area; TH: thickness; AIH: Autoimmune Hyperthyroidism; AIT: Autoimmune Hypothyroidism.

After obtaining the final set of instrumental variables, we applied the Mendelian Randomization Pleiotropy RESidual Sum and Outlier (MR-PRESSO) test to remove potential outliers again prior to conducting each Mendelian randomization analysis. We checked in dbSNP (www.ncbi.nlm.nih.gov/snp) to see whether these SNPs were associated with potential risk factors. The instrumental variables were refined by excluding SNPs associated with cerebral cortical structure and those identified as outliers. The study flowchart is presented in Fig. 1.

### 2.4 MR analysis and sensitivity analysis

This study adopted three Mendelian randomization (MR) approaches to mitigate the interference of consistency bias and pleiotropy, namely the inverse variance weighted (IVW) method, MR-Egger method, and weighted median method. Among the three MR analytical approaches, the IVW method served as the primary analysis strategy, while the MR-Egger method and weighted median method were used to supplement and validate the results derived from the IVW method. These two supplementary methods are capable of generating more robust and reliable estimates under less restrictive assumptions, albeit at the expense of partial precision, which is specifically reflected by the widening of confidence intervals.

Causal estimates were primarily derived using the IVW method (weighted regression through the origin). The weighted median method, robust to up to 50% invalid instruments, provided complementary estimates. To assess the robustness of significant findings, we examined horizontal pleiotropy via the MR-Egger intercept test and leave-one-out analysis, and quantified heterogeneity using Cochran’s Q statistic.

All analyses were performed using R software (version 4.3.2) with the TwoSampleMR (version 0.6.5) and ggses (version 1.6.5) packages. For global-level tests, a two-sided P-value of less than 0.05 was considered significant. In the case of region-level analyses, a P-value of less than 0.05 was regarded as significant.

## 3 Results

In the process of refining the genetic variables used for predicting hyperthyroidism, we identified potential pleiotropic associations of candidate SNPs with confounders. Based on this systematic assessment, the variant rs2476601 was specifically eliminated from consideration. Given that the autoimmune conditions may independently affect brain structure, rs2476601 was excluded to avoid potential horizontal pleiotropy. For predicting hypothyroidism, rs1990760 and rs7574865 were excluded because they were associated with gene expression in the cerebral cortex. These exclusions were performed to minimize the risk of confounding due to pleiotropy. Ultimately, 10 SNPs were used for genetic prediction of hyperthyroidism, while 161 SNPs were used for hypothyroidism prediction. The *F* statistics of these genetic tools are greater than the commonly chosen value of 10, indicating strong instrument performance (Tan, et al.,2023). We identified one overlapping SNP (rs11571297) that was used as an instrumental variable for both hyperthyroidism and hypothyroidism. In the leave-one-one-out sensitivity analysis that accounted for the aforementioned exclusions, the causal effect remained statistically significant and its direction unchanged. Although this SNP slightly influenced the effect size, it did not alter the overall conclusion of the study.

**Fig. 2:**
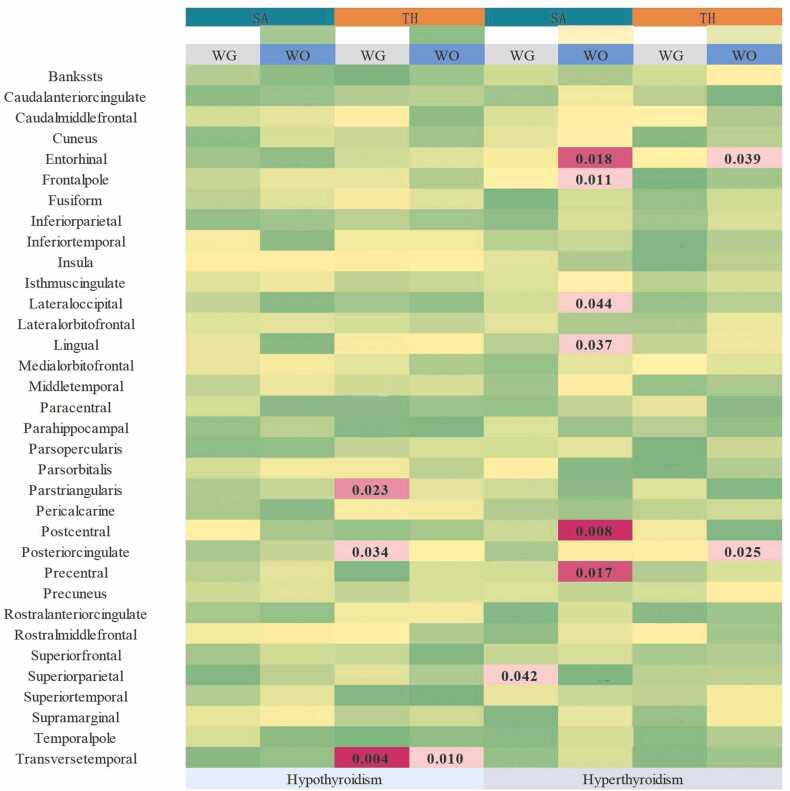
Based on the IVW estimates of the effects of immune hyperthyroidism and hypothyroidism on cortical structures as defined by cerebral cortical surface area and thickness measured by MRI. The color of each block represents the P value derived from the IVW of each MR analysis. SA: Surficial area; TH: thickness; WG: with global weighting; WO: without global weighting.

**Fig. 3:**
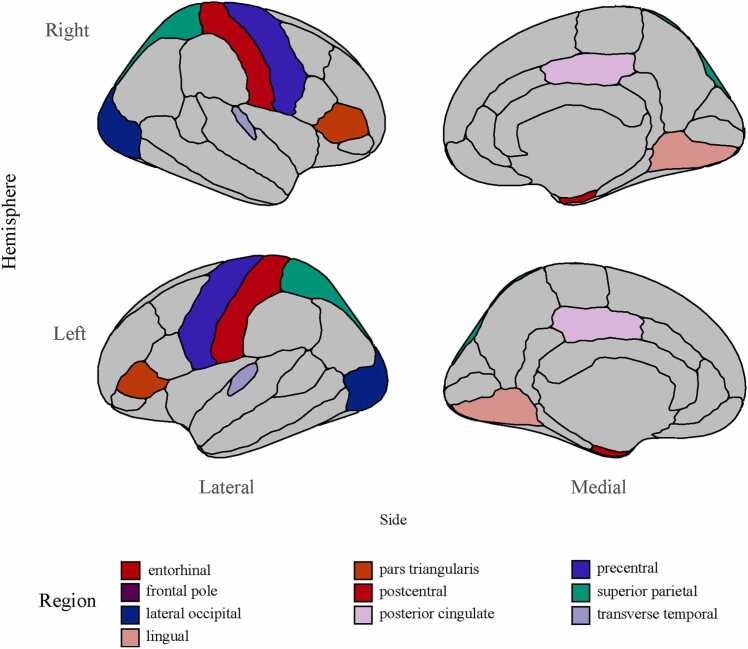
Left and Right Cerebral Hemisphere. The affected areas of the left and right hemispheres of the brain are shown separately on each hemisphere and their lateral and medial sides, distinguished by different colors, while the unaffected areas are displayed in gray.

We conducted a comprehensive MR study on the global surface area and cortical thickness and 34 functional brain regions with or without global weighting, using genetic prediction of hypothyroidism and hyperthyroidism (Fig. 2). We identified several brain regions that showed causal associations with genetically predicted thyroid function (Fig. 3). At the global level, we found no causal relationship between thyroid function and surface area and cortical thickness (Table 1). Hyperthyroidism showed no causal relationship with global surface area and cortical thickness (β_SA_=−364.75, SE_SA_=214.60, P_SA_=0.089; β_TH_=0.002, SE_TH_=0.001, P_TH_=0.17), while predicted hypothyroidism showed no causal relationship with global surface area and cortical thickness (β_SA_=145.91, SE_SA_=193.43, P_SA_=0.45; β_TH_=−0.0007, SE_TH_=0.001, P_TH_=0.56).

**Table 1:** Mendelian randomization estimates from Hyperthyroidism and Hypothyroidism.

**Exposure**	**Exposure**	**Outcome**	**IVW-derived P value**	**β(95% Confidence intervals)**	**Cochran’s Q-derived P value**	**MR-Egger intercept derived P value**
**Hyper**	**Hyperthyroidism**	SA of postcentral without global weighted	**0.008**	−16.360(−28.410 to −4.309)	0.43	0.30
		SA of frontalpole without global weighted	**0.011**	−1.195(−2.118 to −0.271)	0.35	0.47
		SA of precentral without global weighted	**0.017**	−16.089(−29.281 to −2.897)	0.96	0.55
		SA of entorhinal without global weighted	**0.018**	−2.576(−4.714 to −0.437)	0.26	0.62
		SA of lateraloccipital without global weighted	**0.044**	−15.787(−31.171 to −0.403)	0.31	0.37
		SA of lingual without global weighted	**0.037**	−10.778(−20.888 to −0.668)	0.73	0.74
		SA of superiorparietal with global weighted	**0.042**	11.360(0.435–22.285)	0.73	0.30
		TH of entorhinal without global weighted	**0.039**	0.009(0.000–0.018)	0.66	0.18
		TH of posteriorcingulate without global weighted	**0.025**	0.004(0.001–0.008)	0.80	0.86
**Hypo**	**Hypothyroidism**	TH of transversetemporal without global weighted	**0.010**	0.006（0.001–0.011)	0.20	0.39
		TH of transversetemporal with global weighted	**0.004**	0.006（0.002–0.010)	0.15	0.25
		TH of parstriangularis with global weighted	**0.023**	−0.003（−0.005 to −0.001)	0.22	0.35
		TH of posteriorcingulate with global weighted	**0.034**	0.003（0.000–0.006)	0.14	0.70

Significant estimate is defined as IVW-derived P < 0.05. Cochran’s Q-derived P value and MR-Egger Intercept-derived P value < 0.05 is significant. IVW, Inverse-variance weighted; TH, thickness; SA, surficial area.

In functional regional analysis, the causal association patterns of hyperthyroidism and hypothyroidism with surface area and cortical thickness differ significantly. In the cohort with hypothyroidism, our findings indicated causal associations solely with the thickness of the cerebral cortex, irrespective of the consideration of global weighting. For patients with hyperthyroidism, the results show that the surface area shows more significant causal associations and the cortical thickness shows fewer causal associations. When global weights were taken into account, the cortical thickness areas of the Parstriangularis (P_TH_=0.023), Posteriorcingulate (P_TH_=0.034), and Transversetemporal (P_TH_=0.004) showed causal associations with hypothyroidism. Simultaneously, the thickness of Transversetemporal (P_TH_=0.010) also showed causal associations when global weights were not considered. In the context of hyperthyroidism, without global weighting, we observed causal associations with the cortical areas of the Entorhinal (P_SA_=0.018), Frontalpole (P_SA_=0.011), Lateraloccipital (P_SA_=0.044), Lingual (P_SA_=0.037), Postcentral (P_SA_=0.008), and Precentral (P_SA_=0.017) regions. Concurrently, the cortical thickness of the Entorhinal (P_TH_=0.039) and Posteriorcingulate (P_TH_=0.025) also showed causal associations. Upon consideration of global weights, the Superiorparietal (P_SA_=0.042) area exhibits susceptibility to alterations in the setting of hyperthyroidism. Within these impacted regions, no signs of heterogeneity or horizontal pleiotropy were detected. For meaningful estimation, Cochran's *q* test, MR-Egger intercept test(Chen, et al.,2021), leave-one-out analysis, and funnel plot were used to evaluate sensitivity, and the results showed no evidence of multi-level specificity. For detailed information, please refer to Supplementary Tables (Table S1-S4) and Supplementary Figures.

## 4 Discussion

The thyroid gland is the largest endocrine gland in the human body, secreting and stimulating thyroid hormones into the blood, which plays an important role in maintaining and regulating the functions of various tissues and organs(Greenhill,2016). Thyroid diseases are closely related to neurological diseases. Hyperthyroidism, hypothyroidism and abnormal thyroid antibodies can all cause adult central nervous system diseases(Chen, et al.,2022; Li, et al.,2022; Figgie Jr, et al.,2024). Among them, there have been systematic reviews on cerebrovascular diseases related to the thyroid gland, but research on the cerebral cortex of thyroid diseases is still scarce(Siu, et al.,2009; Sheu, et al.,2010; Wollenweber, et al.,2013).

Using the GWAS datasets, we made a breakthrough in conducting the analysis of the effects of immune hyperthyroidism and immune hypothyroidism on the surface area and thickness of the cerebral cortex(Grasby, et al.,2020; Kurki, et al.,2023). The research findings indicate that both autoimmune hypothyroidism and autoimmune hyperthyroidism are capable of bringing about alterations in the area and thickness of the cerebral cortex. Brain magnetic resonance imaging (MRI) might be applied in the early diagnosis of neuropsychiatric disorders in patients with thyroid dysfunction(Krausz, et al.,2004). The underlying mechanism associated with the link between thyroid function and changes in brain function should be further investigated.

Research has demonstrated the critical role thyroid hormones play in brain development, including neuronal migration, myelination, and synaptic formation. Furthermore, these hormones are crucial for maintaining brain homeostasis and regulating mood, cognition, and behavior(de Escobar, et al.,2004). The causal associations of autoimmune hypothyroidism with the thickness of the cerebral cortex were predominantly distributed in areas such as Parstriangularis, Posteriorcingulate, Superiorparietal, and Transversetemporal. Among them, Transversetemporal is the most exceptional, as its thickness increases irrespective of whether the global weighted are taken into account. The causal associations of autoimmune hyperthyroidism with the cerebral cortex were somewhat more complex, primarily involving the surface area of the cerebral cortex. The involved regions comprise Entorhinal, Frontalpole, Lateraloccipital, Lingual, Postcentral, Precentral, and Superiorparietal. The area of the remaining six regions, with the exception of Superiorparietal, is decreased. Moreover, the results for the Entorhinal and Posteriorcingulate regions indicate that their thickness is increased.

The frontal lobe is a part of the anterior portion of the brain cortex, which contains multiple different areas. It plays an essential role in cognitive and behavioral control, such as decision-making, working memory, emotional regulation, social behavior and language processing. Posterior cingulate is affected by hyperthyroidism or hypothyroidism, which mainly involve neural networks related to memory, consciousness, and emotion, and correspond to the clinical symptoms of thyroid disease patients. Adults afflicted with autoimmune hypothyroidism tend to present milder symptoms. A number of researchers postulate that the cognitive impairment ensuing from hypothyroidism in adults is primarily linked to brain tissue hypoxia caused by a decreased blood supply. Individuals within this age cohort may encounter a comprehensive cognitive deterioration encompassing language function, logical ability, and memory. Some may even manifest symptoms such as drowsiness(Rovet,2014). Patients with hypothyroidism also exhibit discrepancies in information processing, sensory integration, attention allocation, and spatial cognition among the Parstrianguillaris, Superior Parietal, and Transversetemporal, which exert definite effects. Some scholars further assert that patients with hypothyroidism have asymmetrical blood flow in the parietal lobe, which may potentially give rise to cognitive impairment and emotional abnormalities, Anti-thyroid autoantibodies have also been found in several specific areas of the human brain(Moodley, et al.,2011; Utku, et al.,2011). Results from studies have shown that patients with hyperthyroidism have abnormal decision-making performance under ambiguous conditions. These deficits may be due to metabolic disturbances in the frontal lobe and limbic system and dopamine dysfunction. Our findings extend the literature on the neuropsychological characteristics of patients with hyperthyroidism. In the future, we will explore the association between hyperthyroidism and cognitive impairment using neuroimaging methods and neurobiological techniques(Yuan, et al.,2015).Hyperthyroidism also exerts an influence on other brain regions, including Entorhinal, Frontalpole, Lateraloccipital, Lingual, Postcentral, Precentral, and Superiorparietal, which are implicated in information processing and integration as well as cognitive functions, particularly in the processing and integration of various sensory information (such as visual, somatosensory, etc.) to support complex cognitive and perceptual tasks. Entorhinal, which is pivotal in memory and spatial navigation, is involved in the integration of sensory information and serves as the primary afferent zone for the hippocampus, being closely linked to memory formation and recall(Mesulam,1998; Poulter, et al.,2018). Patients with hyperthyroidism demonstrate abnormalities in both the surface area and thickness of the Entorhinal region.

In order to produce reliable estimates in MR studies, it is crucial to carefully select appropriate genetic tools. In addition to selecting robust genetic tools with a high *F*-statistic (>10) for clustering SNPs associated with the results, it is important to consider the traits associated with each SNP and mitigate the impact of potential confounding factors(Bi, et al.,2024). To improve the accuracy of our analysis and increase the likelihood of identifying significant estimates, we adopted two sets of genetic tools to represent thyroid function in our study.

The structural alterations in specific brain regions identified in this study hold significant neuroanatomical implications. First, most of the brain regions affected by thyroid dysfunction belong to key functional networks: the default mode network (e.g., posterior cingulate), the executive control network (e.g., frontal pole, pars triangularis), and the sensorimotor network (e.g., precentral and postcentral gyri, transverse temporal gyrus). These networks play central roles in cognitive processing, emotional regulation, and sensory integration. Notably, the posterior cingulate cortex, as a key hub of the default mode network, is critically involved in self-referential thinking, episodic memory retrieval, and consciousness regulation. Its structural alterations may explain common symptoms in thyroid disease patients, such as "brain fog," memory decline, and attentional deficits. Second, the involvement of the transverse temporal gyrus (auditory cortex) and lingual gyrus (visual association cortex) suggests that thyroid dysfunction may affect sensory processing pathways, aligning with clinical observations of altered sensitivity to sensory stimuli in thyroid disease patients. Importantly, hypothyroidism and hyperthyroidism exhibit distinct patterns of causal association: hypothyroidism is primarily associated with cortical thickness, whereas hyperthyroidism is more frequently associated with surface area. As a hypothesis-generating speculation, this discrepancy might reflect different underlying mechanisms—such as effects on dendritic density versus neuronal number—but this requires direct validation in future studies.

Our findings, derived from a genetic instrumental variable analysis, must be interpreted within the broader context of thyroid hormone action on the central nervous system. Thyroid hormones exert their effects primarily through genomic pathways mediated by nuclear receptors (TRα and TRβ), regulating the expression of a vast array of genes involved in neurodevelopment, metabolism, and myelination. The regional specificity observed in our study—whereby hyperthyroidism and hypothyroidism affect distinct sets of cortical areas—may reflect differential expression patterns of these receptor isoforms or region-specific cofactor availability. For instance, the prominent involvement of sensorimotor regions (precentral, postcentral) and visual association areas (lingual, lateral occipital) in hyperthyroidism could be linked to the high metabolic demands and rich mitochondrial density in these regions, making them particularly susceptible to the hypermetabolic state induced by thyrotoxicosis. Conversely, the impact on regions within the default mode network (posterior cingulate) in both conditions highlights a potential common pathway affecting self-referential and introspective cognitive processes, possibly via modulation of serotoninergic or noradrenergic neurotransmission, which are known to be influenced by thyroid status and are densely represented in these networks. This mechanistic speculation aligns with the clinical phenomenology but requires direct validation. Future studies integrating transcriptomic data from post-mortem human brain atlases with our genetic findings could help map the spatial correlation between thyroid hormone receptor expression and the brain regions identified here as causally susceptible, offering a direct neurobiological link between genetic risk, molecular pathways, and macroscopic structural outcomes.

The findings of this study carry substantial clinical significance. First, the identified vulnerable brain regions may serve as early biomarkers for thyroid-related cognitive dysfunction. For instance, increased thickness of the posterior cingulate cortex and decreased surface area of the postcentral gyrus could potentially serve as neuroimaging markers for screening neuropsychiatric complications in thyroid disease patients. Second, these findings underscore the importance of placing greater emphasis on the assessment and intervention of neurocognitive function in the management of thyroid diseases. Clinicians should not only monitor thyroid hormone levels but also regularly evaluate patients' cognitive function and emotional states, with neuroimaging examinations conducted when necessary. Furthermore, the study results provide insights for developing novel therapeutic strategies. For example, cognitive training or non-invasive brain stimulation techniques targeting neuroplasticity in specific brain regions may help alleviate cognitive symptoms in thyroid disease patients. Future research should delve into the following directions: First, conducting longitudinal studies to track changes in brain structure before and after thyroid function treatment, evaluating the effects of thyroid hormone replacement therapy or antithyroid medications on brain structure. Second, integrating multimodal neuroimaging techniques (e.g., fMRI, DTI) to explore the relationships between brain structural changes and functional connectivity or white matter integrity. Third, investigating underlying mechanisms through animal models or cellular experiments to elucidate the molecular pathways through which thyroid hormones influence neurodevelopment and plasticity in specific brain regions. Fourth, expanding the study population to examine differences in brain structural alteration patterns among patients of different ages, genders, and disease subtypes, thereby providing a basis for personalized treatment. These studies will contribute to establishing a more comprehensive theoretical framework of thyroid-brain interactions, ultimately improving the quality of life and long-term prognosis of thyroid disease patients.

In summary, current research indicates that thyroid function plays a critical role in brain development and function, and alterations in thyroid hormone levels may contribute to the development of cognitive and psychiatric disorders. Future studies should aim to better understand the underlying pathophysiological mechanisms linking thyroid function and neuropsychiatric disorders in hypothyroidism patients, or identify novel treatment modalities that may prevent or alleviate neuropsychiatric and cognitive impairment associated with hypothyroidism.

Several limitations should be acknowledged. First, outcomes were analyzed without multiple testing correction; findings should be considered hypothesis-generating. Second, despite using PhenoScanner and MR-PRESSO, residual horizontal pleiotropy cannot be completely ruled out. Third, this study only covered autoimmune hyperthyroidism and hypothyroidism, not subclinical or non-autoimmune causes. Fourth, both FinnGen and ENIGMA data were derived primarily from European populations; generalizability to other ancestries requires caution. Fifth, brain cortical structure data were from cross-sectional MRI measurements, which cannot capture dynamic changes over time; longitudinal studies are needed. Sixth, GWAS summary statistics did not allow stratification by sex, age, or thyroid hormone levels.

## 5 Conclusion

This groundbreaking MRI analysis provides a comprehensive examination of the association between thyroid dysfunction and cortical brain structure. The results reveal significant causal associations, indicating that both hypothyroidism and hyperthyroidism are causally associated with cortical thickness. These findings open new possibilities for using brain MRI as an early diagnostic tool for identifying neuropsychiatric disorders in individuals with thyroid dysfunction. Further investigations are needed to explore the underlying mechanisms that link thyroid function with changes in brain functionality.

## Funding

This study was supported by the 10.13039/501100001809National Natural Science Foundation of China (No. 82004138); Key Project of Natural Science Foundation of Nanjing University of Chinese Medicine (No. XZR2024015); Excellent Young Doctor Training Program of Jiangsu Province Hospital of Chinese Medicine (No. 2023QB0126).

## CRediT authorship contribution statement

**Jianhuai Chen:** Writing – review & editing, Writing – original draft, Visualization, Validation, Supervision, Software, Resources, Project administration, Methodology, Investigation, Formal analysis, Data curation, Conceptualization. **Jianxin Shi:** Writing – review & editing, Writing – original draft, Visualization, Validation, Supervision, Software, Resources, Project administration, Methodology, Investigation, Formal analysis, Data curation, Conceptualization. **Heng Zhao:** Writing – review & editing, Writing – original draft, Visualization, Validation, Supervision, Software, Resources, Project administration, Methodology, Investigation, Funding acquisition, Formal analysis, Data curation, Conceptualization. **Ruifeng Wang:** Writing – review & editing, Writing – original draft, Visualization, Validation, Supervision, Software, Resources, Project administration, Methodology, Investigation, Formal analysis, Data curation, Conceptualization.

## Conflicts of Interest

The authors declare no conflict of interest.
