# Supplementary material for: Causal effect of thyroid function on cortical brain structure: A two-sample Mendelian randomization study
Source: IBRO Neurosci Rep. 2026 Jun 15;21:114–21. doi: 10.1016/j.ibneur.2026.06.010 (PMC13293755; doi:10.1016/j.ibneur.2026.06.010)
Supplement: Supplementary Table S1 — Supplementary material [file mmc1.docx]

**Supplementary Figures**

**1. Forest plots of causal effects of autoimmune hyperthyroidism on cortical surface area**

| 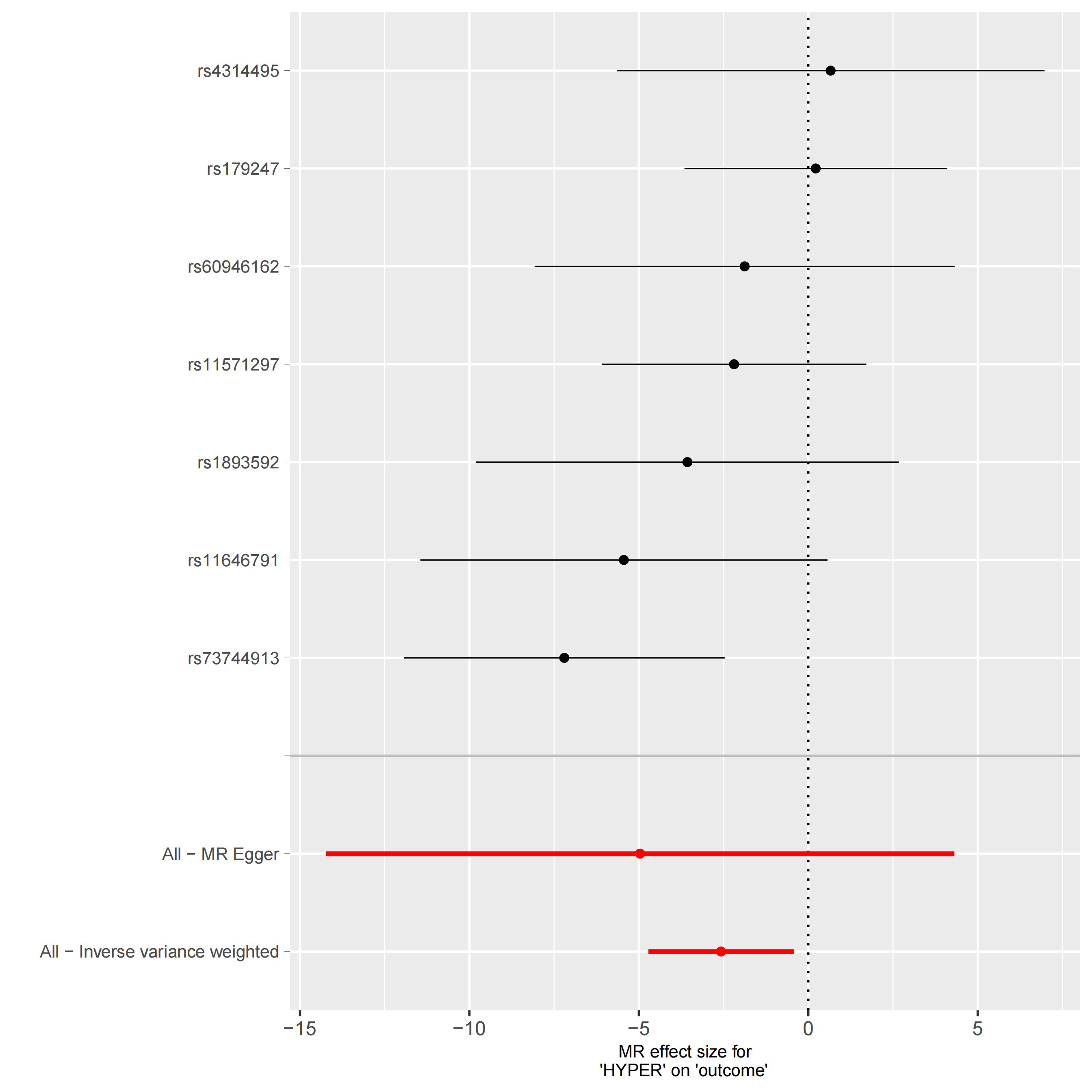 | 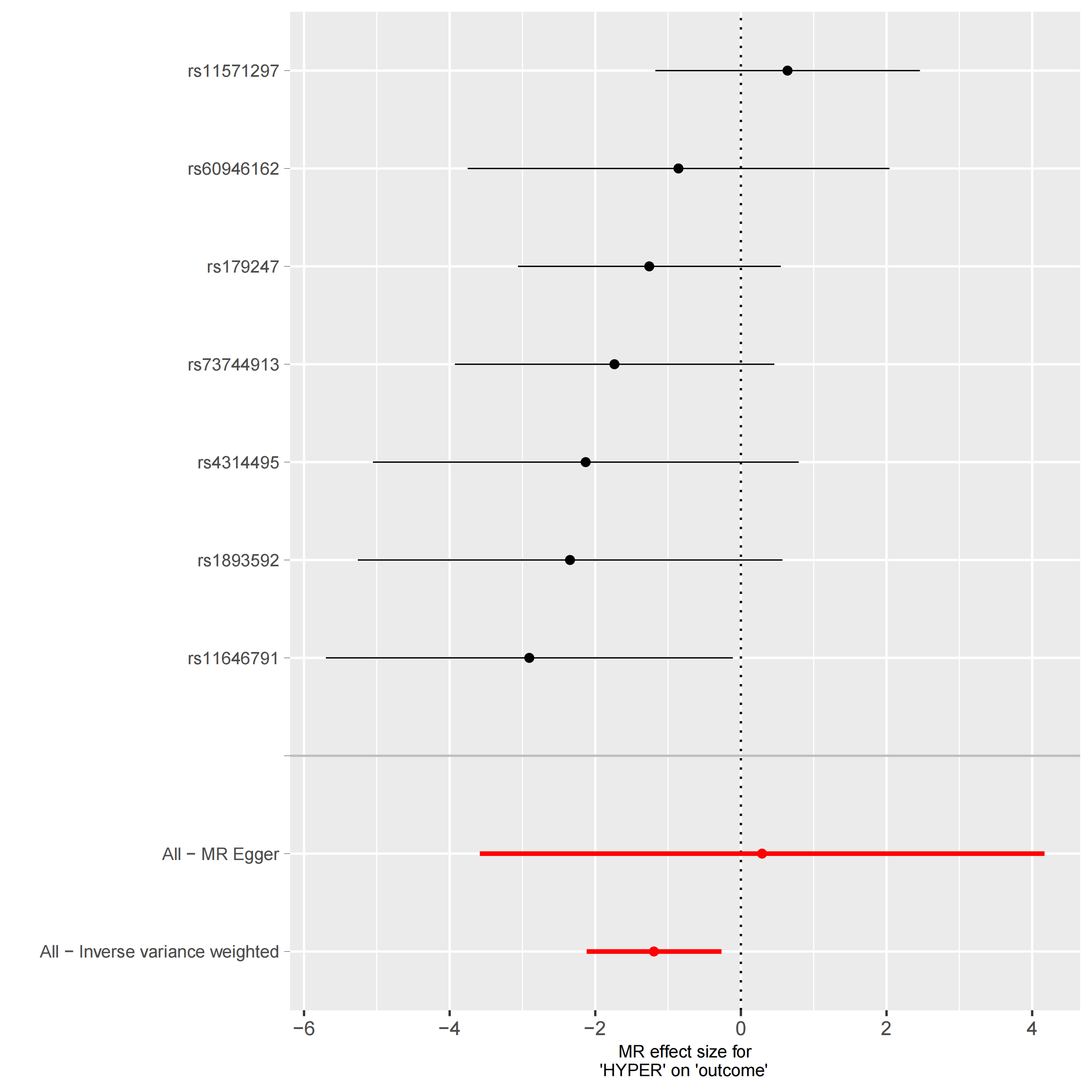 | 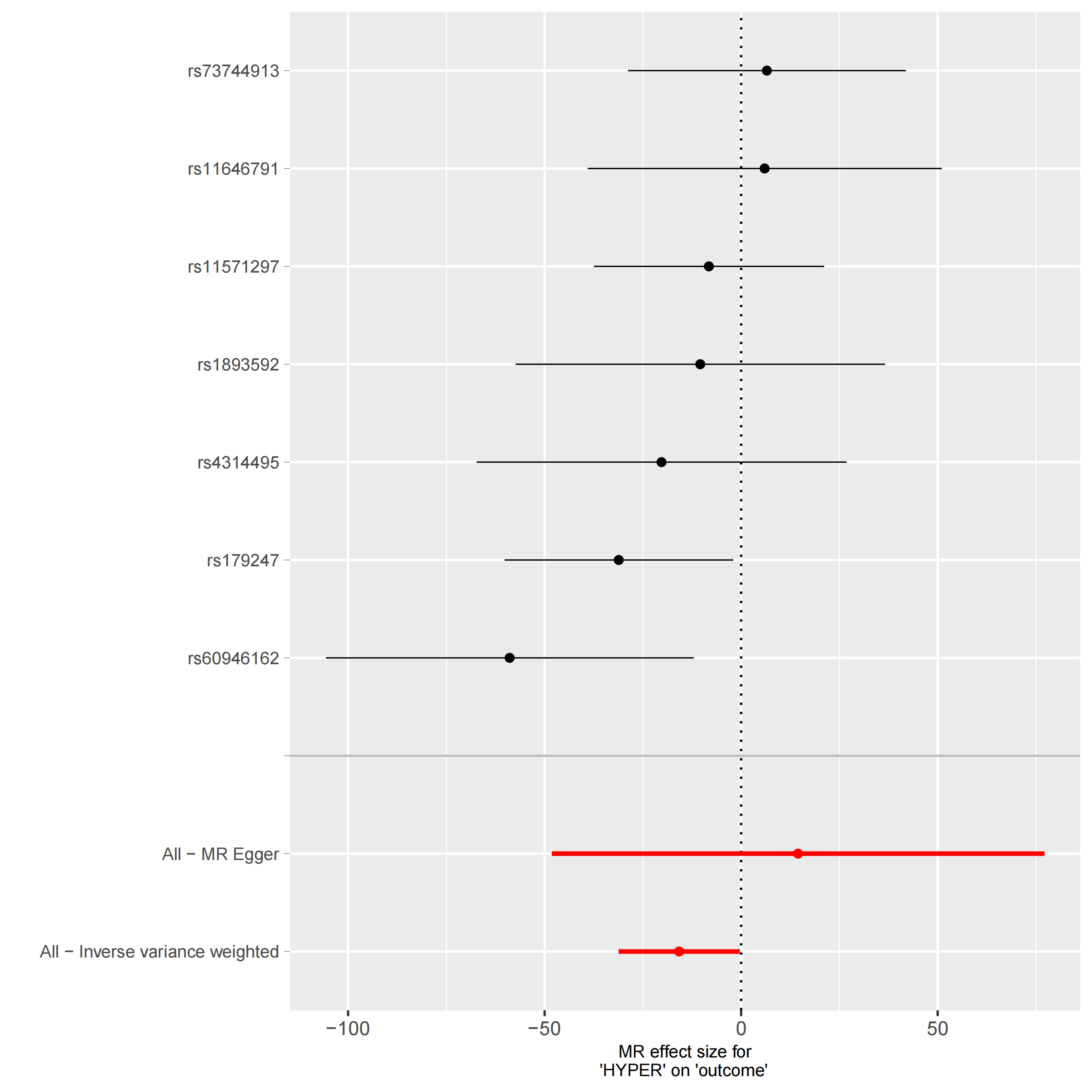 |
| --- | --- | --- |
| Without_global_weighting entorhinal | Without_global_weighting frontalpole | Without_global_weighting lateraloccipital |
| 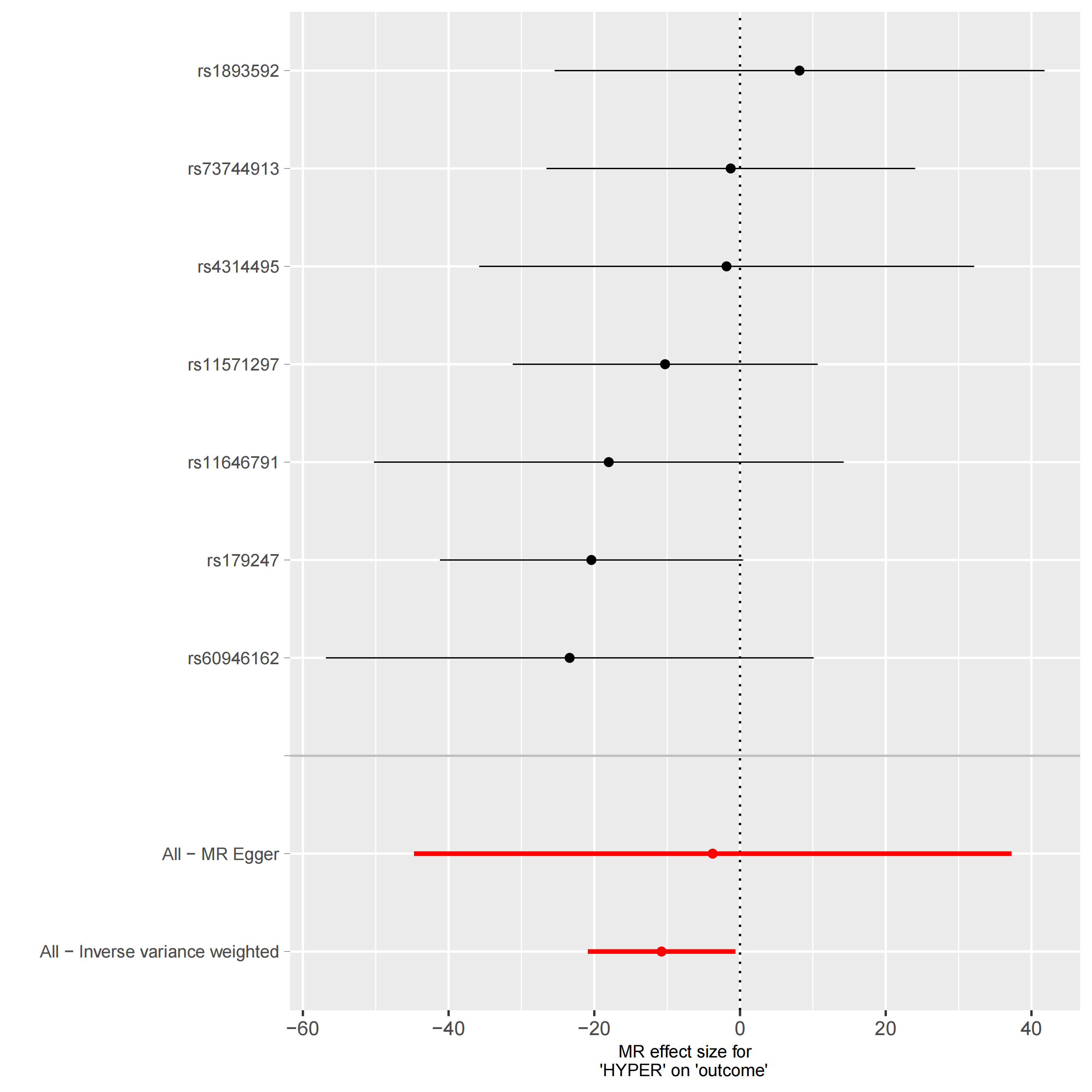 | 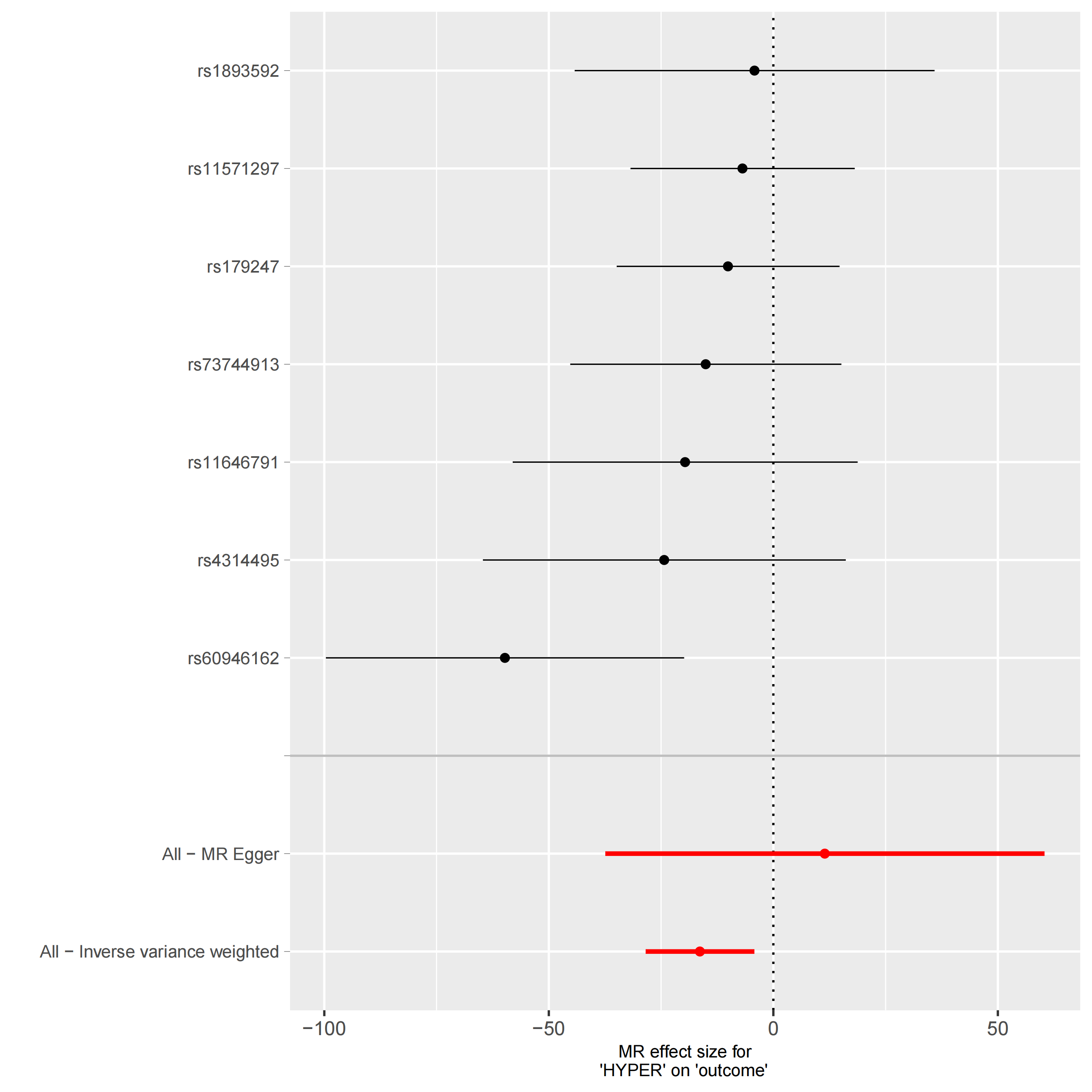 | 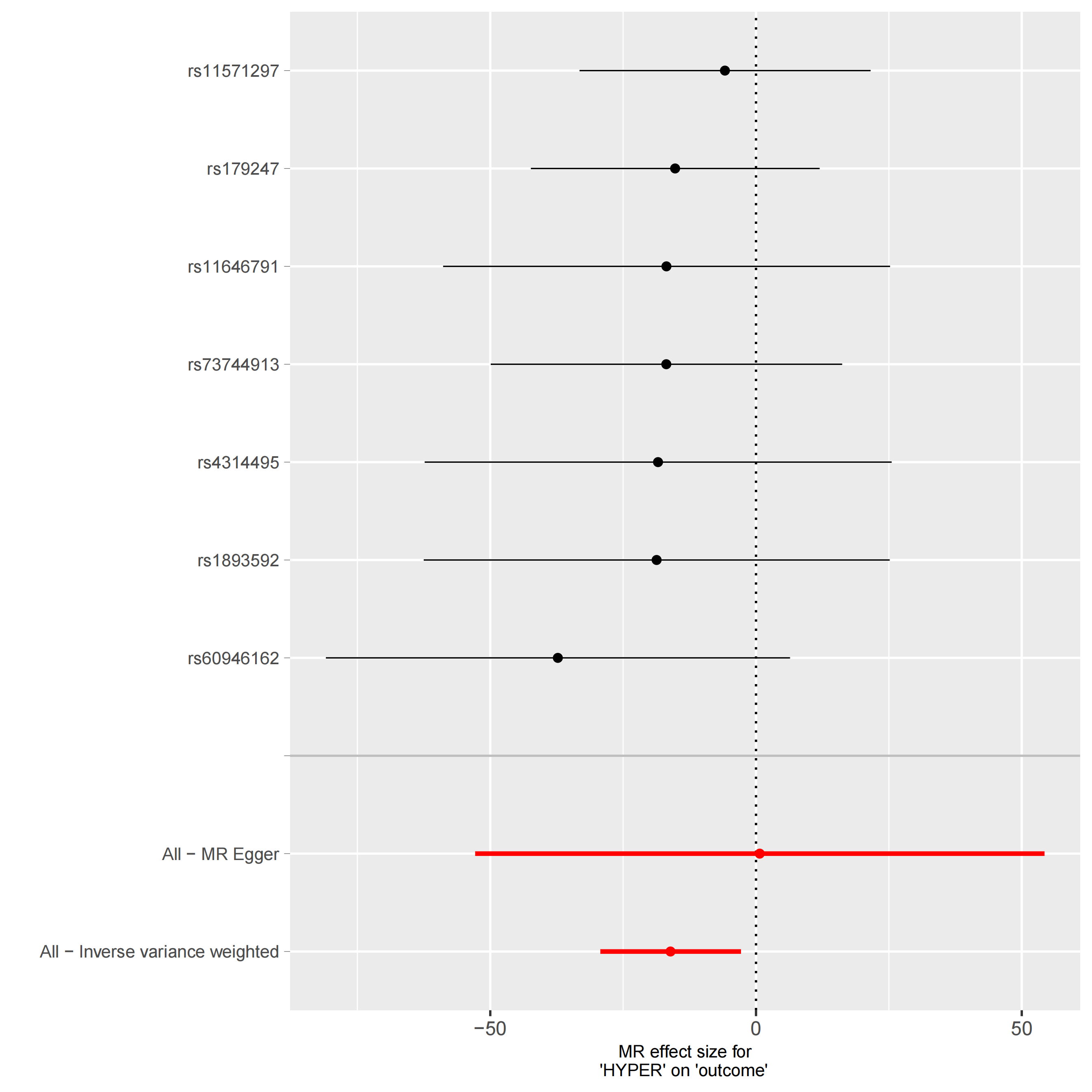 |
| Without_global_weighting lingual | Without_global_weighting postcentral | Without_global_weighting precentral |
|  | 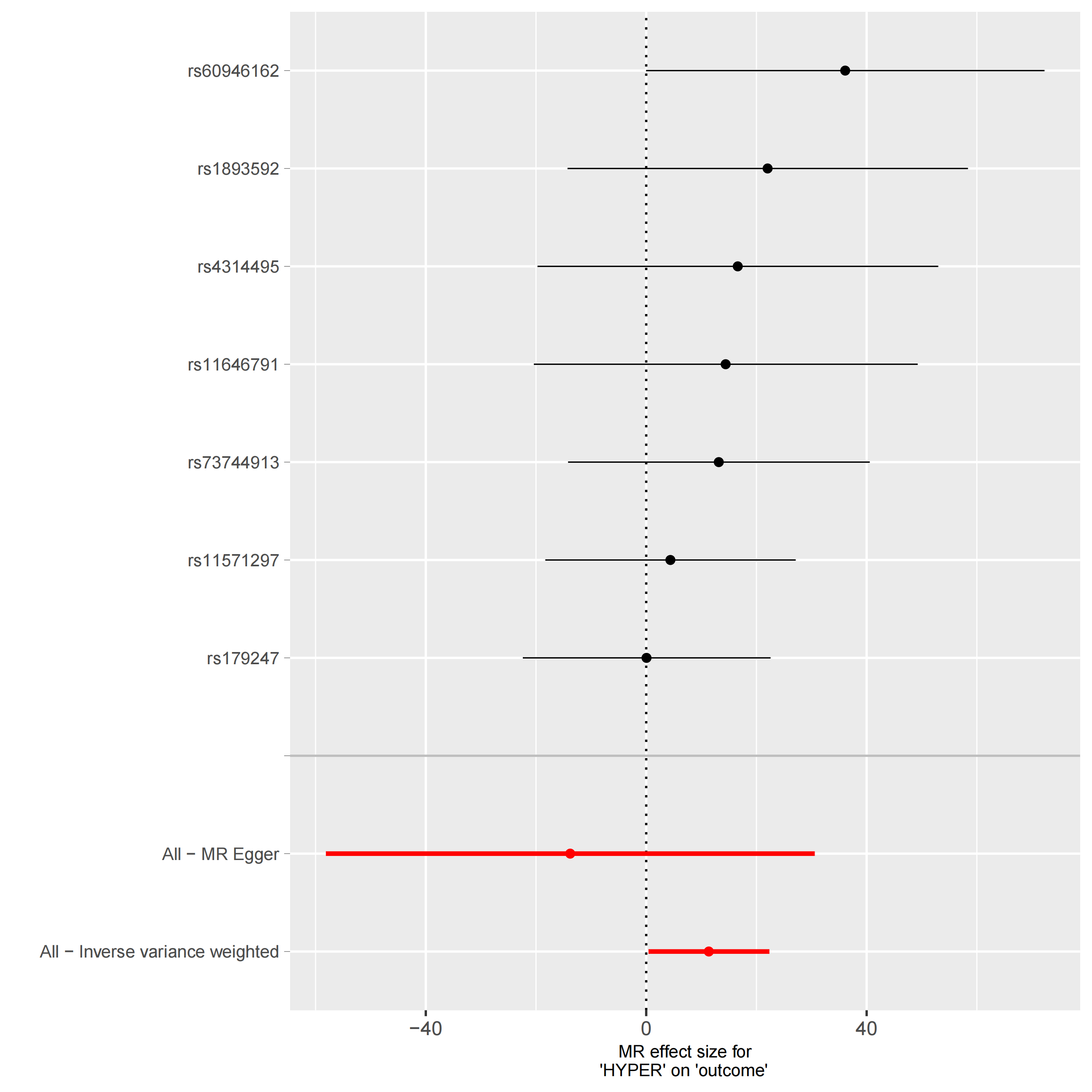 |  |
|  | With_global_weighting superiorparietal |  |

**2. Forest plots of causal effects of autoimmune hyperthyroidism on cortical thickness**

| 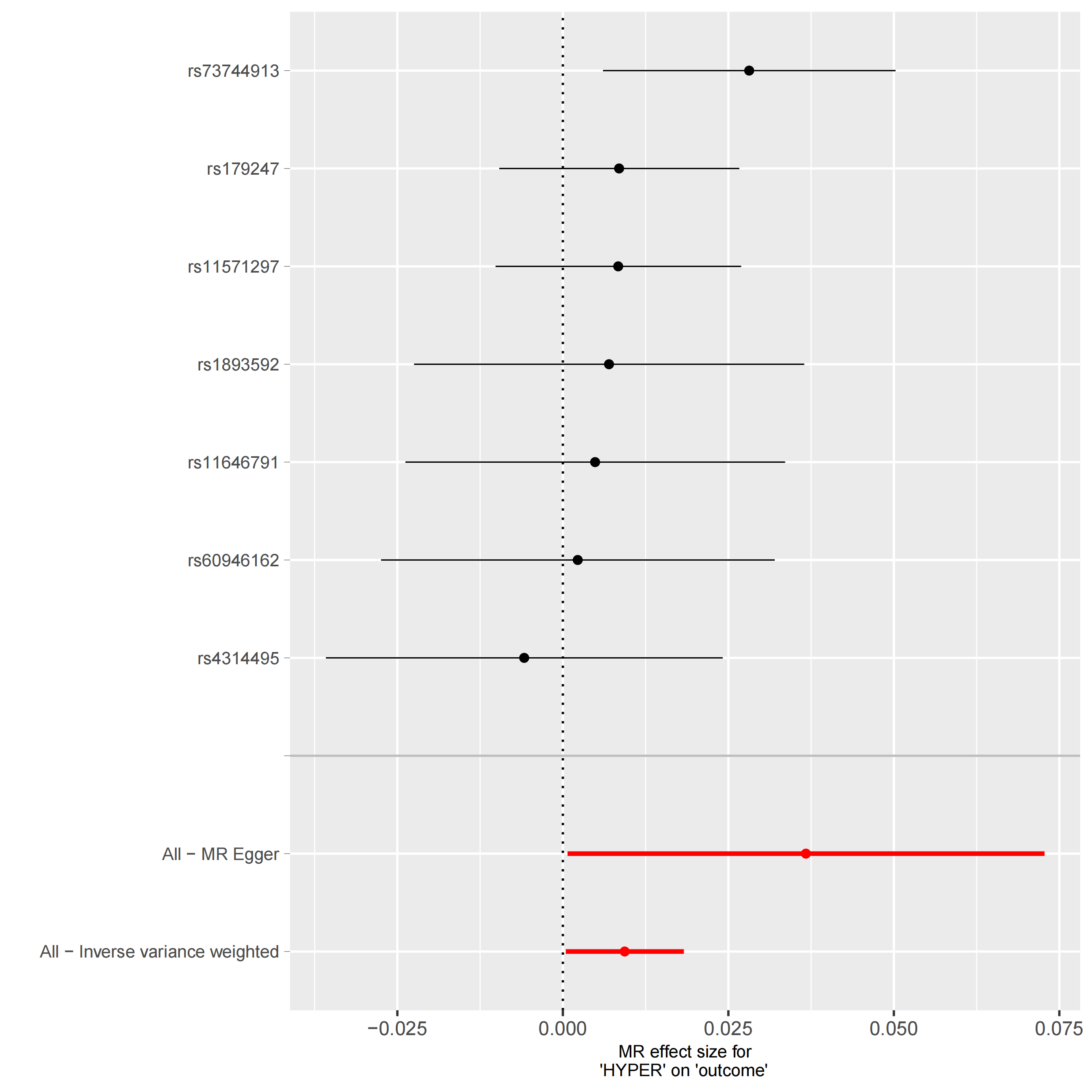 |  | 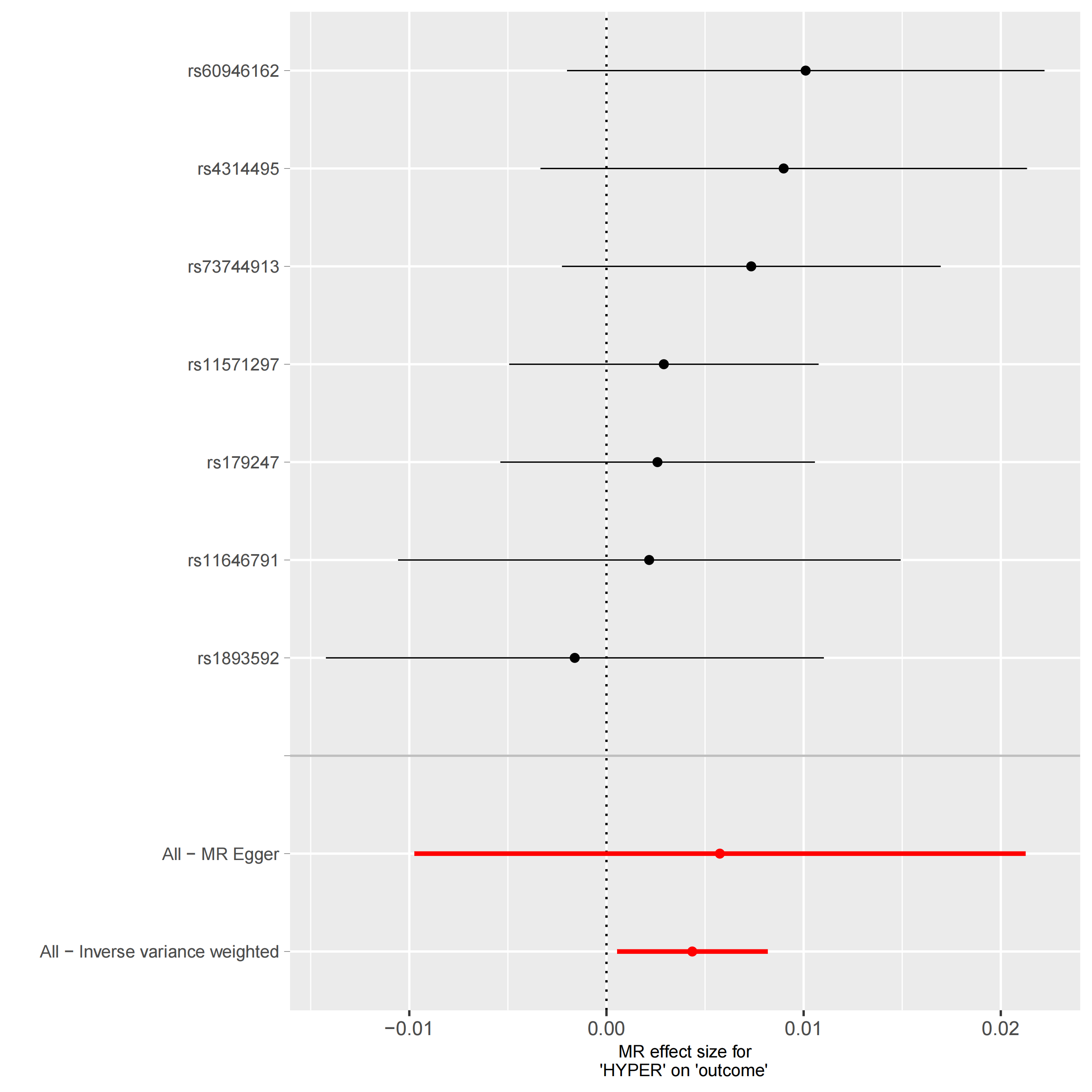 |
| --- | --- | --- |
| Without_global_weighting entorhinal |  | Without_global_weighting posteriorcingulate |

**3. Forest plots of causal effects of autoimmune hypothyroidism on cortical thickness**

| 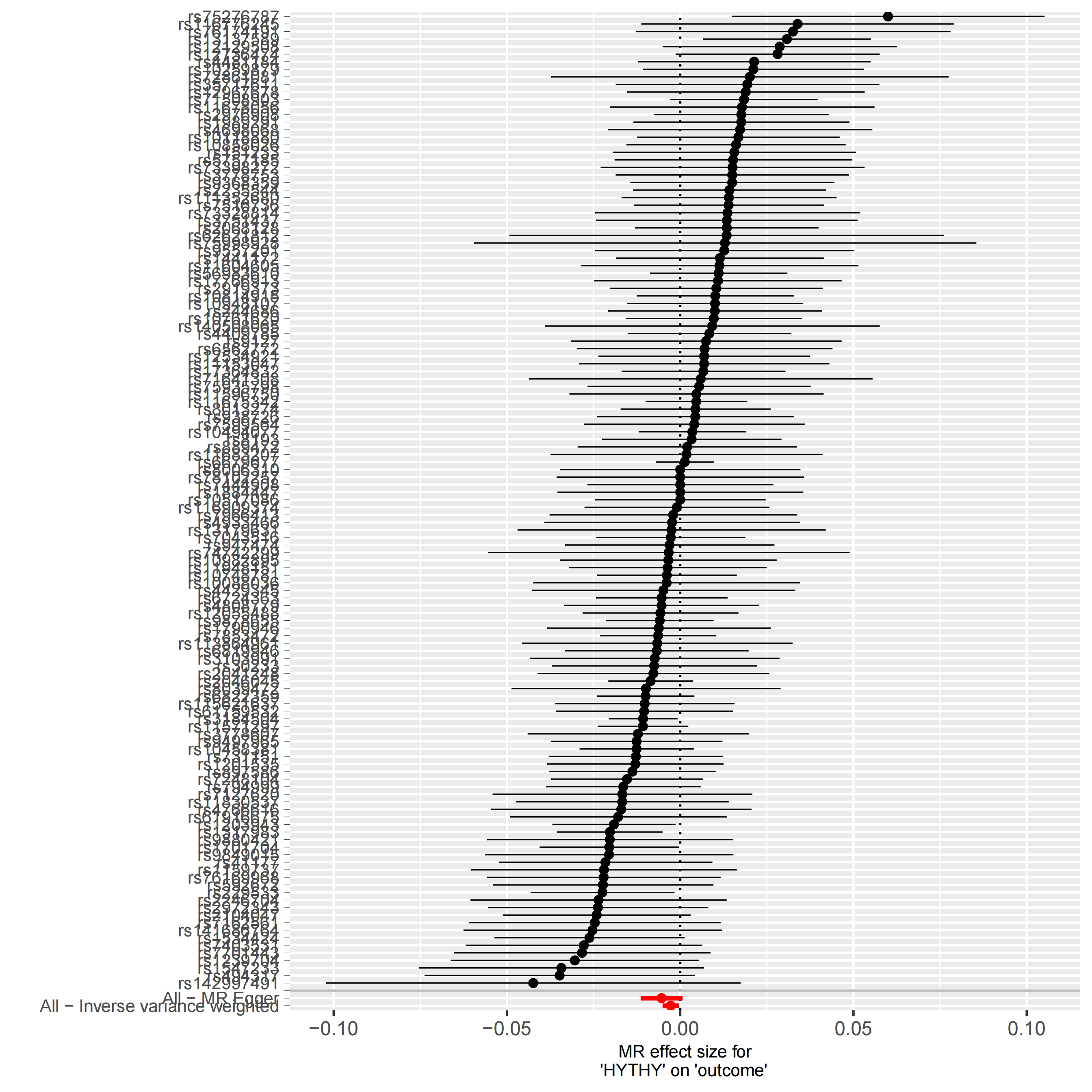 | 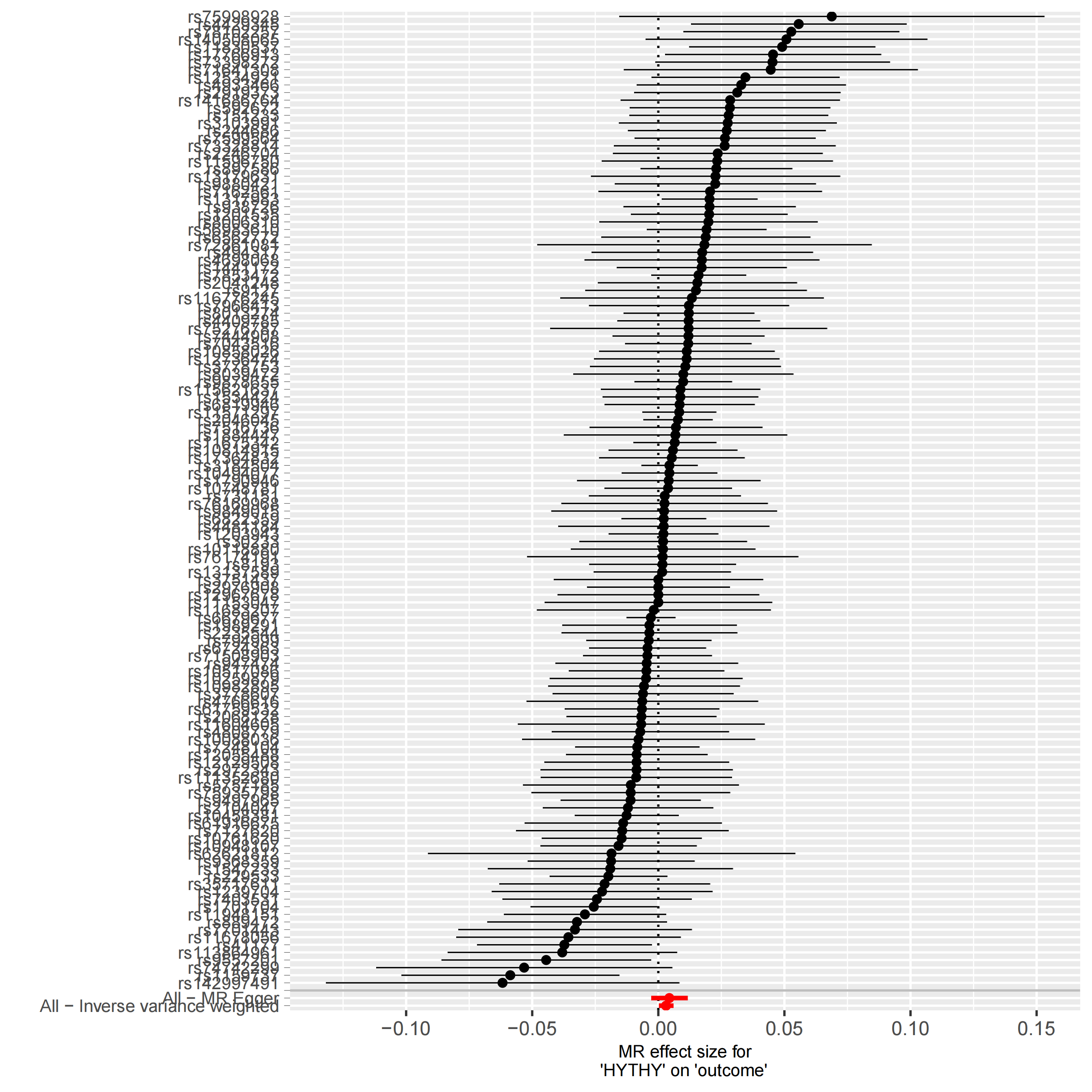 | 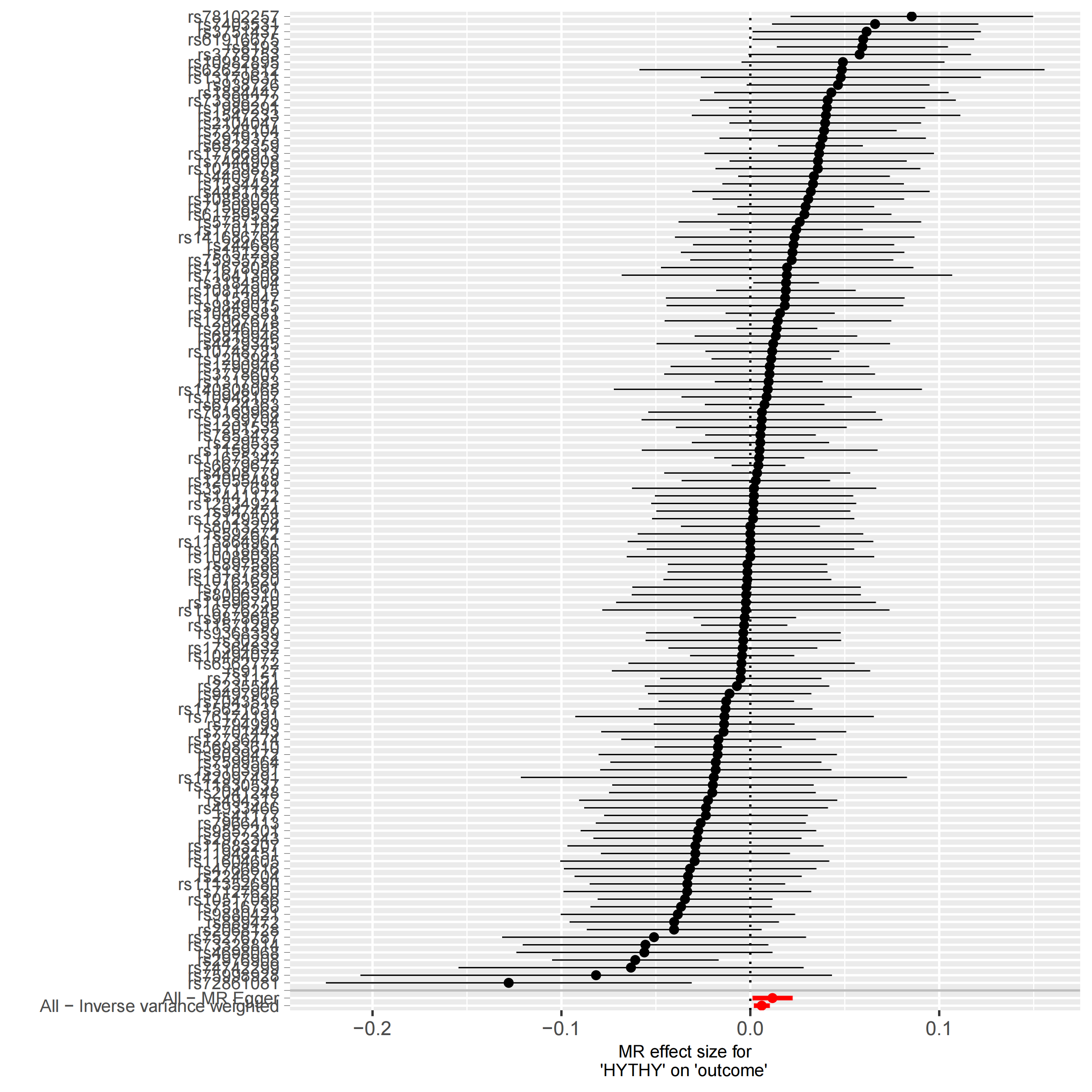 |
| --- | --- | --- |
| With_global_weighting parstriangularis | With_global_weighting posteriorcingulate | With_global_weighting transversetemporal |
|  | 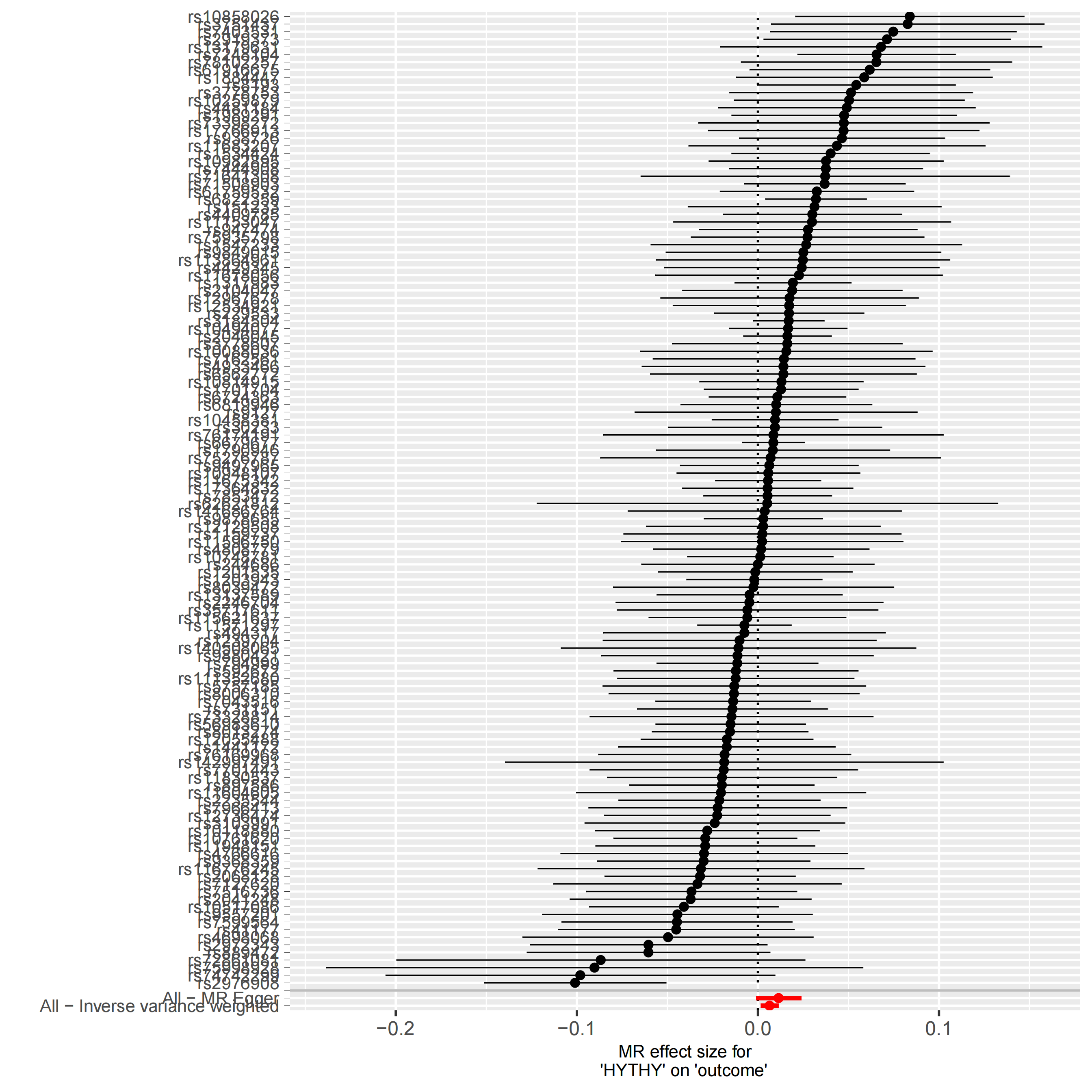 |  |
|  | Without_global_weighting transversetemporal |  |

**4. Funnel plots of causal effects of autoimmune hyperthyroidism on cortical surface area**

| 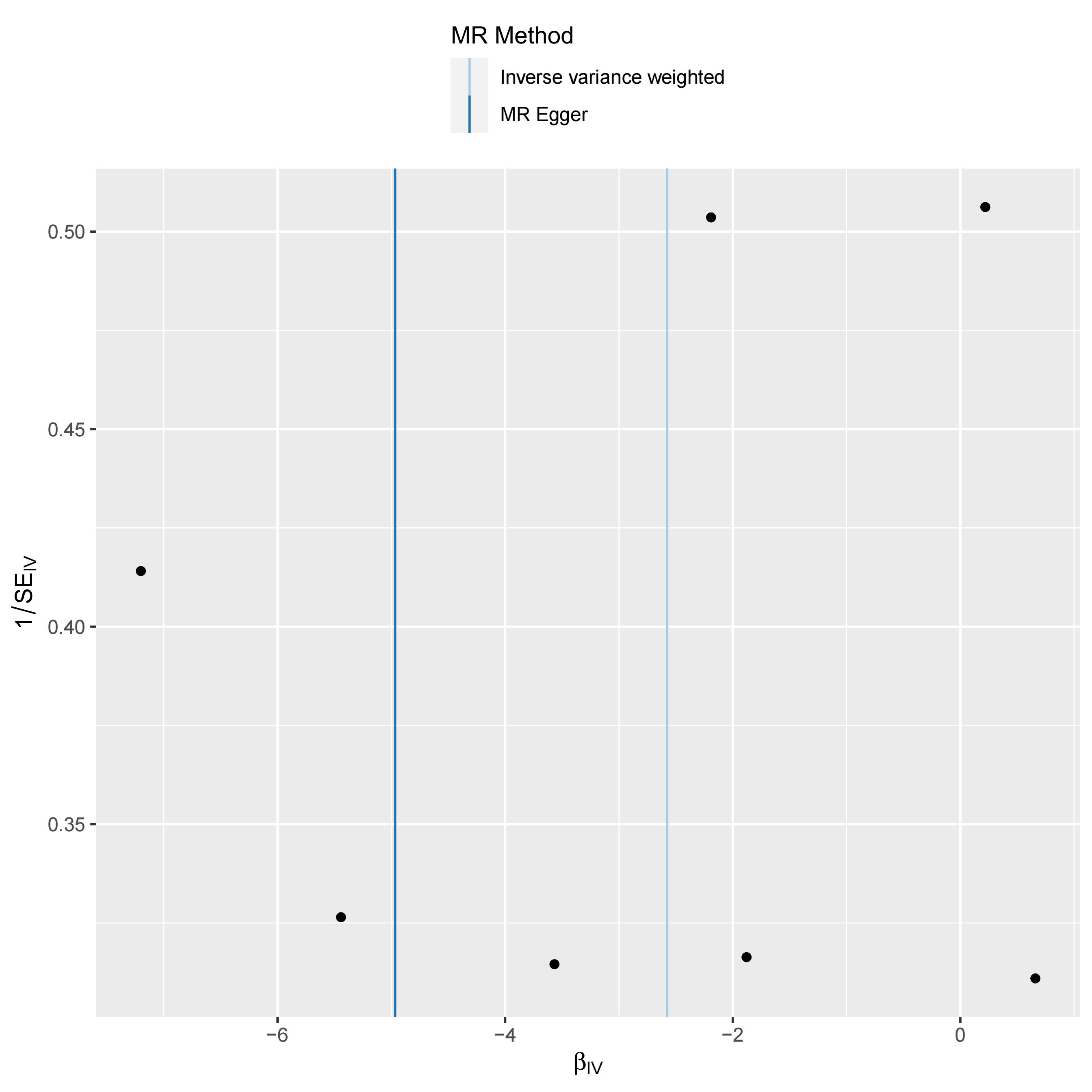 | 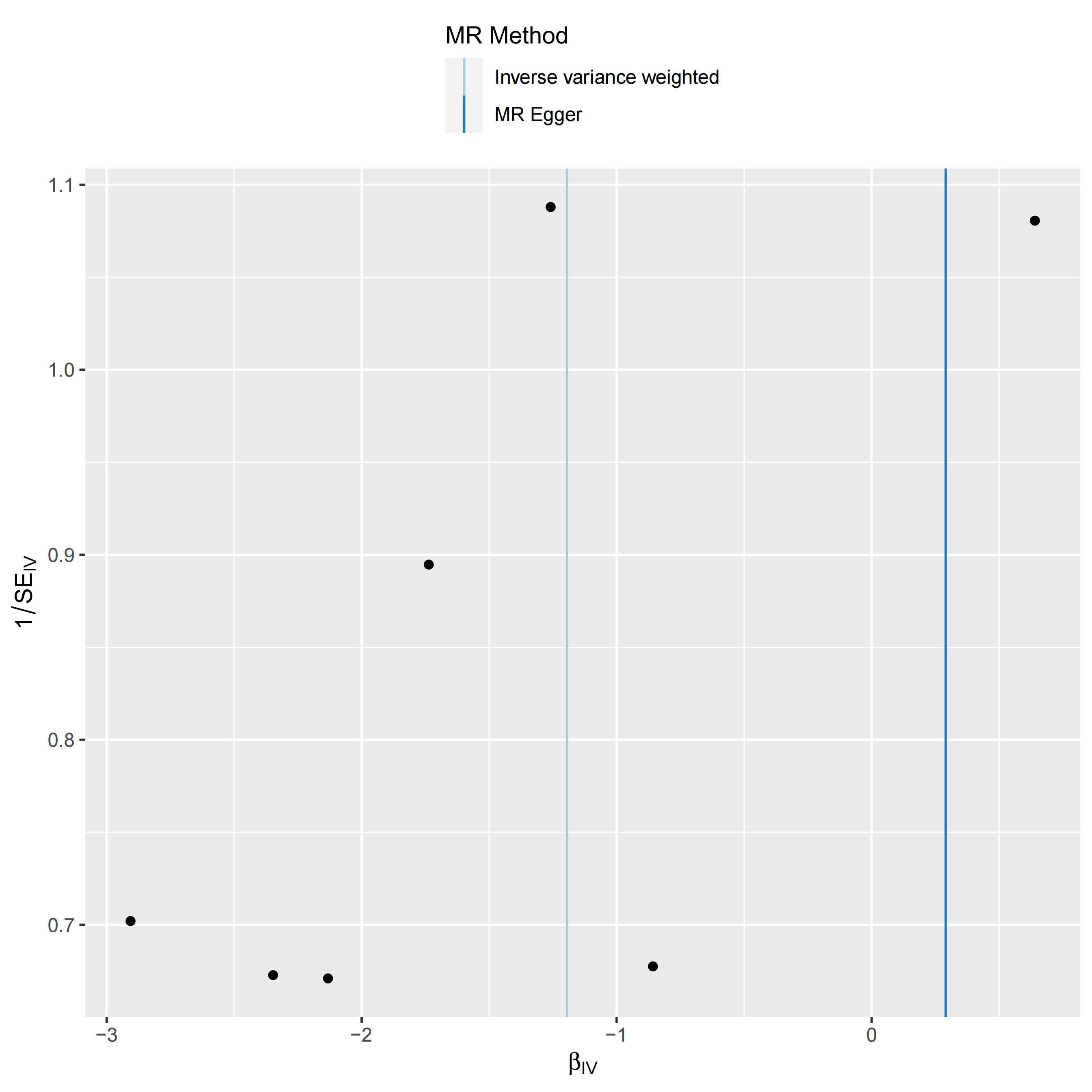 | 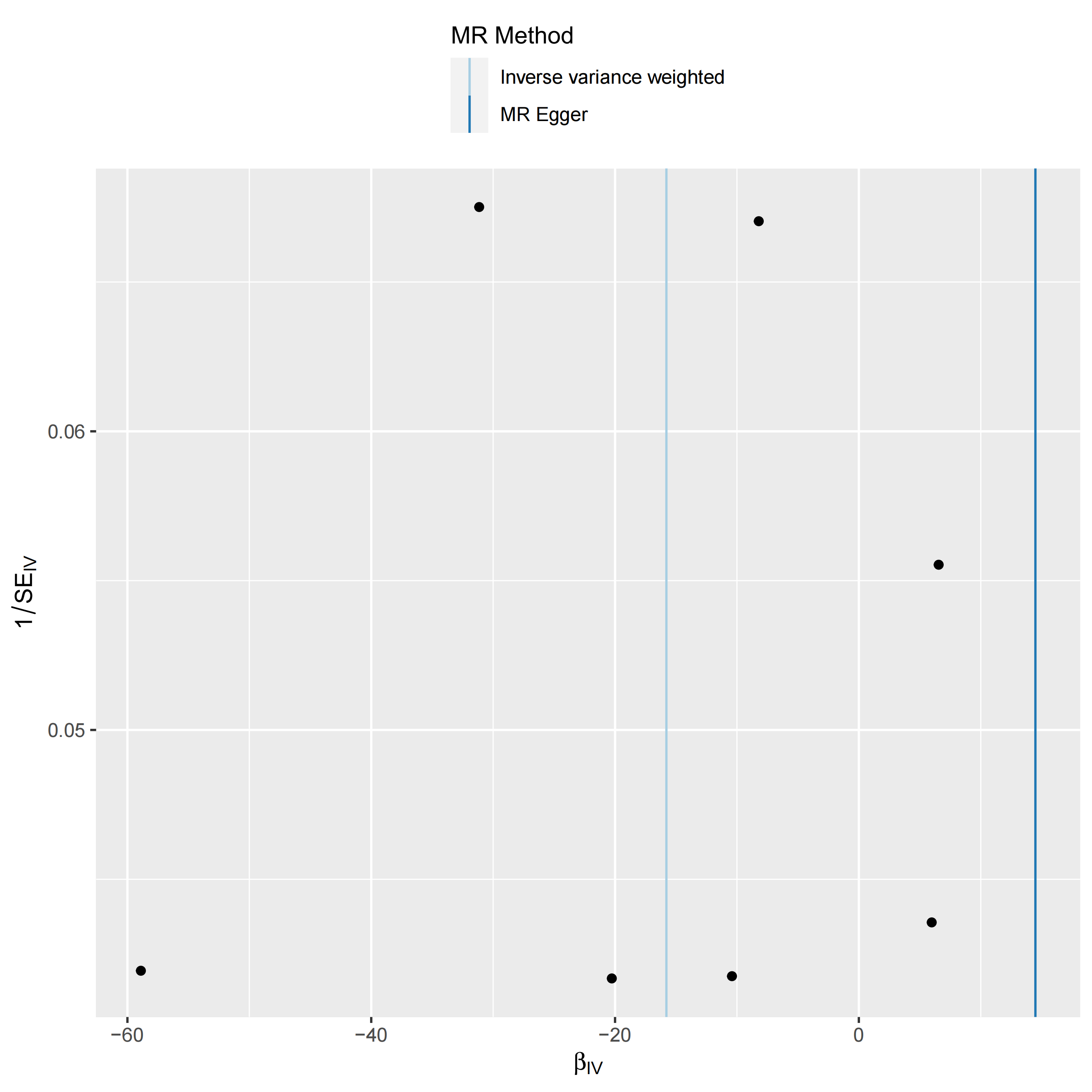 |
| --- | --- | --- |
| Without_global_weighting entorhinal | Without_global_weighting frontalpole | Without_global_weighting lateraloccipital |
| 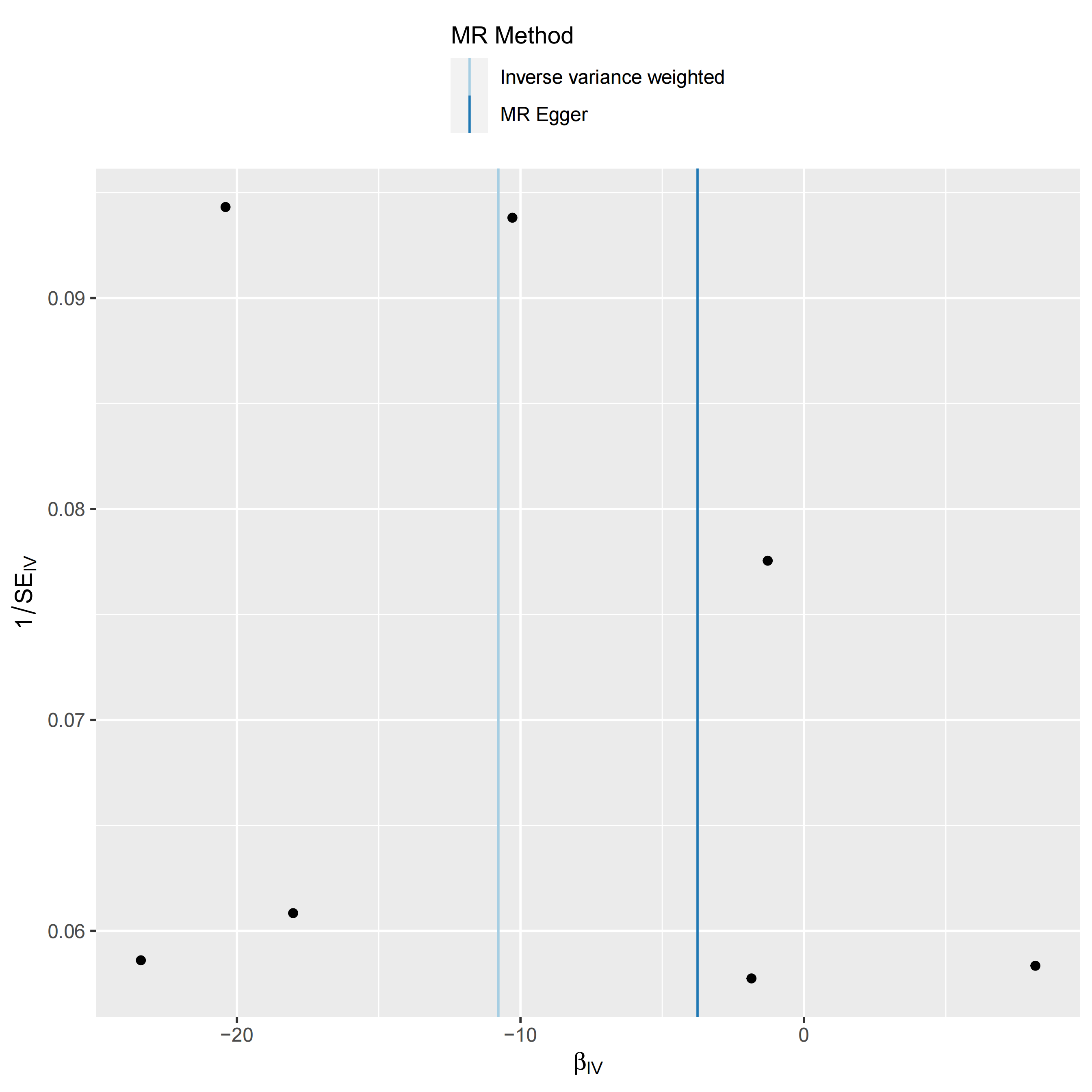 | 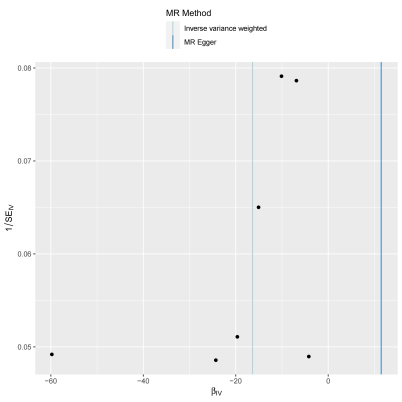 | 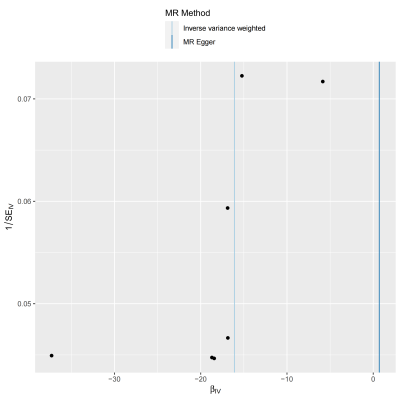 |
| Without_global_weighting lingual | Without_global_weighting postcentral | Without_global_weighting precentral |
|  | 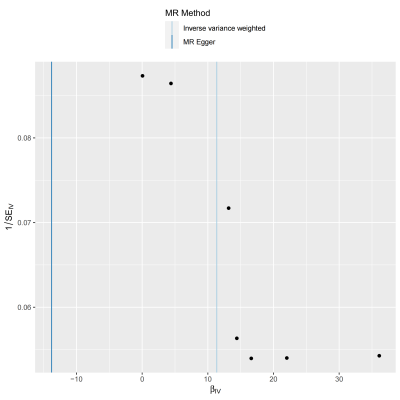 |  |
|  | With_global_weighting superiorparietal |  |

**5. Funnel plots of causal effects of autoimmune hyperthyroidism on cortical thickness**

| 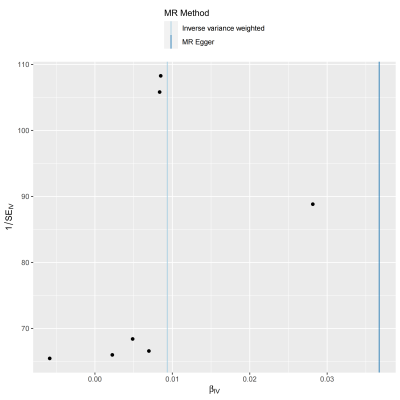 |  | 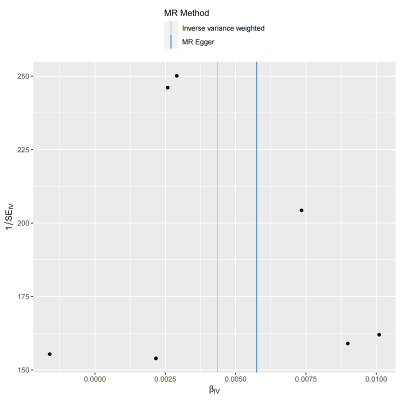 |
| --- | --- | --- |
| Without_global_weighting entorhinal |  | Without_global_weighting posteriorcingulate |

**6. Funnel plots of causal effects of autoimmune hypothyroidism on cortical thickness**

| 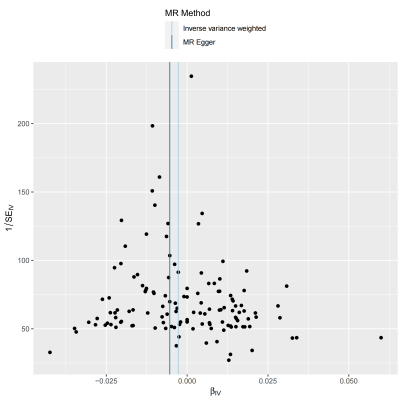 | 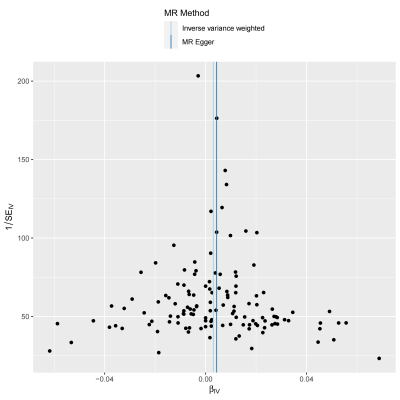 | 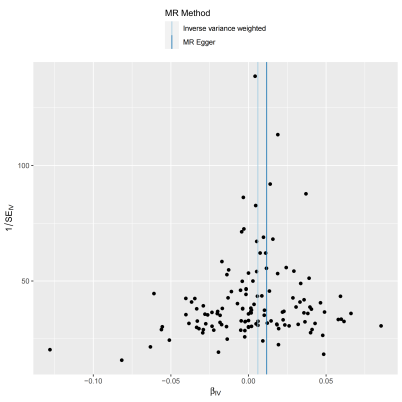 |
| --- | --- | --- |
| With_global_weighting parstriangularis | With_global_weighting posteriorcingulate | With_global_weighting transversetemporal |
|  | 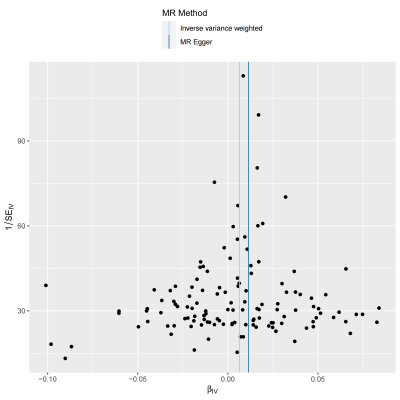 |  |
|  | Without_global_weighting transversetemporal |  |

**7. Leave-one-out plots of causal effects of autoimmune hyperthyroidism on cortical surface area**

| 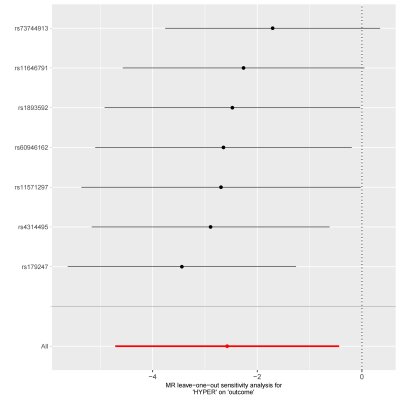 | 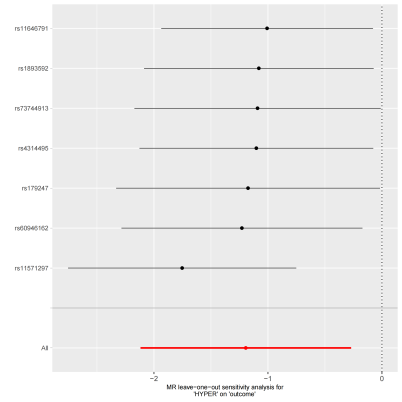 | 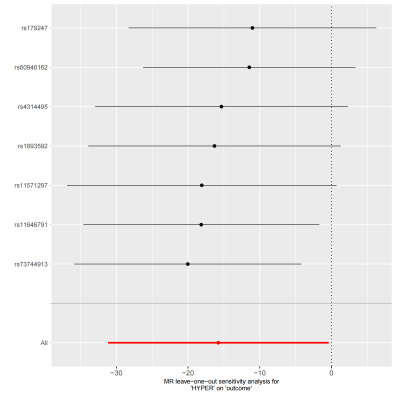 |
| --- | --- | --- |
| Without_global_weighting entorhinal | Without_global_weighting frontalpole | Without_global_weighting lateraloccipital |
| 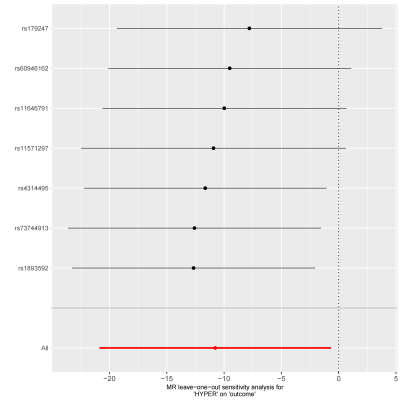 | 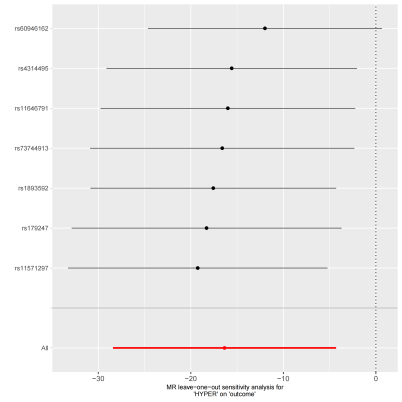 | 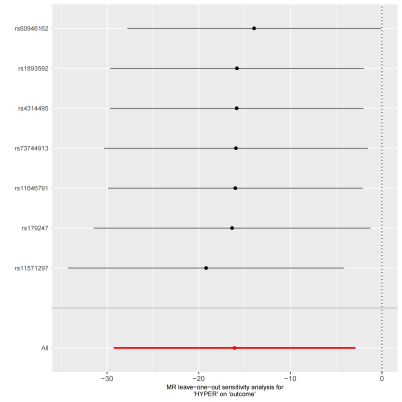 |
| Without_global_weighting lingual | Without_global_weighting postcentral | Without_global_weighting precentral |
|  | 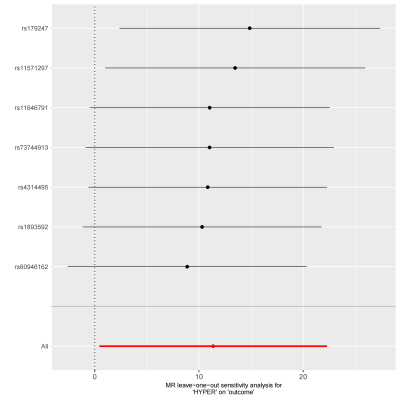 |  |
|  | With_global_weighting superiorparietal |  |

**8. Leave-one-out plots of causal effects of autoimmune hyperthyroidism on cortical thickness**

| 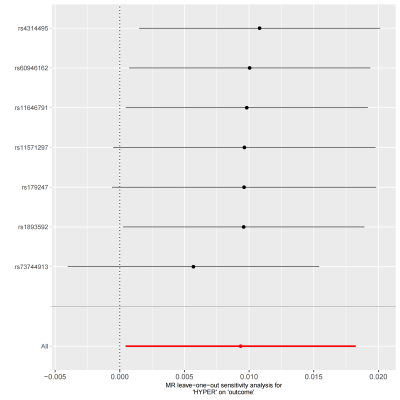 |  | 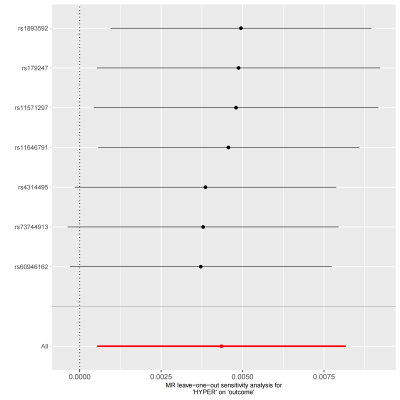 |
| --- | --- | --- |
| Without_global_weighting entorhinal |  | Without_global_weighting posteriorcingulate |

**9. Leave-one-out plots of causal effects of autoimmune hypothyroidism on cortical thickness**

| 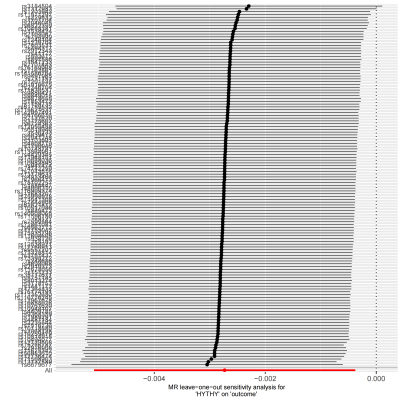 | 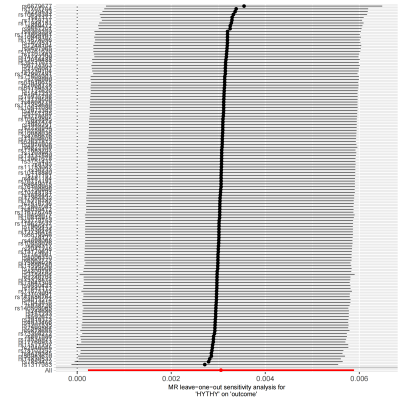 | 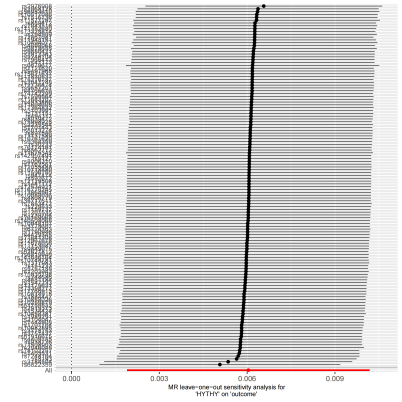 |
| --- | --- | --- |
| With_global_weighting parstriangularis | With_global_weighting posteriorcingulate | With_global_weighting transversetemporal |
|  | 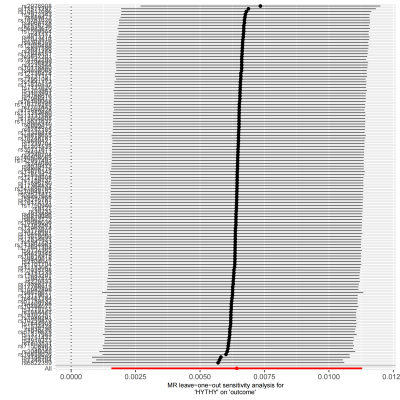 |  |
|  | Without_global_weighting transversetemporal |  |
